# Host-Erythrocytic Sphingosine-1-Phosphate Regulates *Plasmodium* Histone Deacetylase Activity and Exhibits Epigenetic Control over Cell Death and Differentiation

**DOI:** 10.1128/spectrum.02766-22

**Published:** 2023-02-06

**Authors:** Raj Kumar Sah, Sakshi Anand, Waseem Dar, Ravi Jain, Geeta Kumari, Evanka Madan, Monika Saini, Aashima Gupta, Nishant Joshi, Rahul Singh Hada, Nutan Gupta, Soumya Pati, Shailja Singh

**Affiliations:** a Special Centre for Molecular Medicine, Jawaharlal Nehru University, New Delhi, India; b School of Natural Sciences, Department of Life Sciences, Shiv Nadar University, Greater Noida, India; National University of Singapore

**Keywords:** malaria, *Plasmodium*, sphingosine kinase, sphingosine 1 phosphate, histone deacetylase, epigenetic regulation, multistage inhibition

## Abstract

The evolution of resistance to practically all antimalarial drugs poses a challenge to the current malaria elimination and eradication efforts. Given that the epigenome of Plasmodium falciparum governs several crucial parasite functions, pharmaceutical interventions with transmission-blocking potential that target epigenetic molecular markers and regulatory mechanisms are likely to encounter drug resistance. In the malaria parasite, histone deacetylases (HDACs) are essential epigenetic modulators that regulate cellular transcriptional rearrangements, notably the molecular mechanisms underlying parasite proliferation and differentiation. We establish “*lipid sequestration*” as a mechanism by which sphingolipids, specifically Sphingosine-1-Phosphate (S1P) (a metabolic product of Sphingosine Kinase 1 [SphK-1]), regulate epigenetic reprogramming in the parasite by interacting with, and modulating, the histone-deacetylation activity of *Pf*HDAC-1, thereby regulating *Plasmodium* pathogenesis. Furthermore, we demonstrate that altering host S1P levels with PF-543, a potent and selective Sphk-1 inhibitor, dysregulates *Pf*HDAC-1 activity, resulting in a significant increase in the global histone acetylation signals and, consequently, transcriptional modulation of genes associated with gametocytogenesis, virulence, and proliferation. Our findings point to a hitherto unrecognized functional role for host S1P-mediated sphingolipid signaling in modulating *Pf*HDAC-1’s enzymatic activity and, as a result, the parasite’s dynamic genome-wide transcriptional patterns. The epigenetic regulation of parasite proliferation and sexual differentiation offers a novel approach for developing host-targeted therapeutics to combat malaria resistance to conventional regimens.

**IMPORTANCE** Sphingolipid is an 18-carbon amino-alcohol-containing lipid with a sphingosine backbone, which when phosphorylated by sphingosine kinase 1 (SphK-1), generates sphingosine-1-phosphate (S1P), an essential lipid signaling molecule. Dysregulation of S1P function has been observed in a variety of pathologies, including severe malaria. The malaria parasite *Plasmodium* acquires a host S1P pool for its growth and survival. Here, we describe the molecular attuning of histone deacetylase-1 (*Pf*HDAC-1), a crucial epigenetic modulator that contributes to the establishment of epigenetic chromatin states and parasite survival, in response to S1P binding. Our findings highlight the host lipid-mediated epigenetic regulation of malaria parasite key genes.

## INTRODUCTION

The COVID-19 pandemic interrupted malaria services, resulting in an increase in cases and fatalities, indicating that malaria could be yet another dreadful consequence of the crisis (1). Moreover, current malaria elimination and eradication efforts are being challenged by the emergence of resistance to virtually all antimalarial medications, particularly artemisinin-based combination regimens, which are the cornerstone of malaria chemotherapy. Once the intra-erythrocytic proliferation of the parasite peaks, there is always a considerable selective pressure for resistance to current medications to emerge. However, following differentiation into non-proliferating gametocytes, the less abundant forms (micro- and macrogametes, ookinetes, oocysts, and sporozoites) are at lower risk of resistance emergence (2). As a result, pleiotropic drugs primarily directed at hepatic- and gametocyte-stage parasites are likely to encounter drug resistance.

The developmental cycle of the malaria parasite is accompanied by dynamic global transcriptional profiles that are highly correlated with genome-wide gene expression patterns (3–6). Despite its early evolutionary position among protozoa, Plasmodium falciparum possesses several layers of genetic and phenotypic diversity, gene flow, and drug resistance loci that have evolved, allowing it to develop a distinct epigenomic landscape of parasite differentiation and transmit malaria. The epigenome of P. falciparum governs several essential parasite processes, including proliferation during asexual development and sexual differentiation. It is well established that P. falciparum is susceptible to chemical scaffolds (‘*epi*-drugs’) shared by multistage active compounds that target its epigenetic machinery, particularly epigenetic modifiers, and prevent the development of asexual parasites and differentiation of gametocytes (7).

The apparent transcriptional divergence between the parasite’s asexual and sexual developmental phases (7, 8) has prompted investigations to develop pharmaceutical interventions with transmission-blocking potential that target molecular signatures, and regulatory mechanisms that are similar in both the development stages of the parasite. In the malaria parasite, histone deacetylases (HDACs) serve as crucial epigenetic modulators, which are transcriptional repressors and corepressors, and contribute to the erasure of epigenetic modifications, the establishment of epigenetic chromatin states, and regulation of heritable changes in gene expression, thus contributing to the parasite survival (9). *Plasmodium falciparum* HDAC (*Pf*HDAC) inhibition has been shown to activate and repress transcriptionally regulated genes throughout the P. falciparum life cycle, thus altering the steady-state level of histone acetylation across the parasite genome, and causing the transcriptional cascade to collapse (9–11). In P. falciparum, HDACs have been divided into 3 classes (I-III), along with an unclassified novel HDAC (12). The solitary class I HDAC (*Pf*HDAC-1), in particular, is essential for stage-specific transcriptional activity and governs developmental events such as schizogony, gametocytogenesis, and hepatocyte invasion (9). Recently, Kanyal et al. demonstrated that phosphorylation of *Pf*HDAC-1 is required for its catalytic activity, and that its abundance and genomic occupancy vary between artemisinin-resistant and sensitive parasites (13). Importantly, *Pf*HDAC-1 downregulation in artemisinin-resistant P. falciparum indicates its importance as a regulator of cellular transcriptional reorganization during the emergence of drug resistance (14). Chemical inhibition of *Pf*HDAC-1 has been reported to result in aberrant gene expression, particularly downregulation of erythrocyte invasion-related genes, resulting in parasite growth retardation, which is consistent with the *Pf*HDAC-1-knockdown phenotype (11). This indicates that *Pf*HDAC-1 is a potential drug target for overcoming multidrug resistance.

It is well established that nuclear sphingosine-1-phosphate (S1P), a metabolic product of sphingosine kinase 1 (SphK-1), regulates the dynamic turnover of histone acetylation and contextual chromatin states, which is linked to epigenetic regulation of gene transcription in response to environmental cues (15). This is attributed to S1P binding to HDACs and inhibiting their functional activity, thus altering states of histone acetylation, and conferring epigenetic regulation of gene expression. Prior studies have shown that P. falciparum imports lipid species, including complex sphingolipids (16–18). As a result, host lipids serve as a reservoir for parasite-mediated salvage. Given the fact that P. falciparum genome does not encode for SphK, we recently established parasite codependency on the endogenous S1P pool of host erythrocytes, and provided molecular insights into the cellular processes regulated by S1P in the parasite (19, 20). We hypothesize that the host-directed S1P regulates epigenetic reprogramming in the parasite by interacting with, and modulating, the histone-deacetylation activity of *Pf*HDAC-1.

In this study, we show that P. falciparum salvages the host S1P, which interacts with and regulates *Pf*HDAC-1 activity in the parasite nucleus, thus determining the expression profile of parasite genes involved in asexual development, gametocytogenesis, and virulence. This indicates rapid repression of a large percentage of the parasite genome by host S1P. The transcriptional status of α-tubulin, in particular, was shown to be significantly altered, implying that α-tubulin expression is a potential transcriptional marker of HDAC-1 suppression in the malarial parasite, consistent with previous studies (9, 21). Furthermore, we establish the role of host S1P in the growth and development of the early sporogonic stages of Plasmodium berghei. Altogether, our findings suggest a hitherto unrecognized functional role of host S1P-mediated sphingolipid signaling in regulating the enzymatic activity of *Pf*HDAC-1 and, consequently, the dynamic genome-wide transcriptional profiles of the parasite.

## RESULTS

### S1P interacts with r*Pf*HDAC-1 *in vitro*.

**FIG 1:**
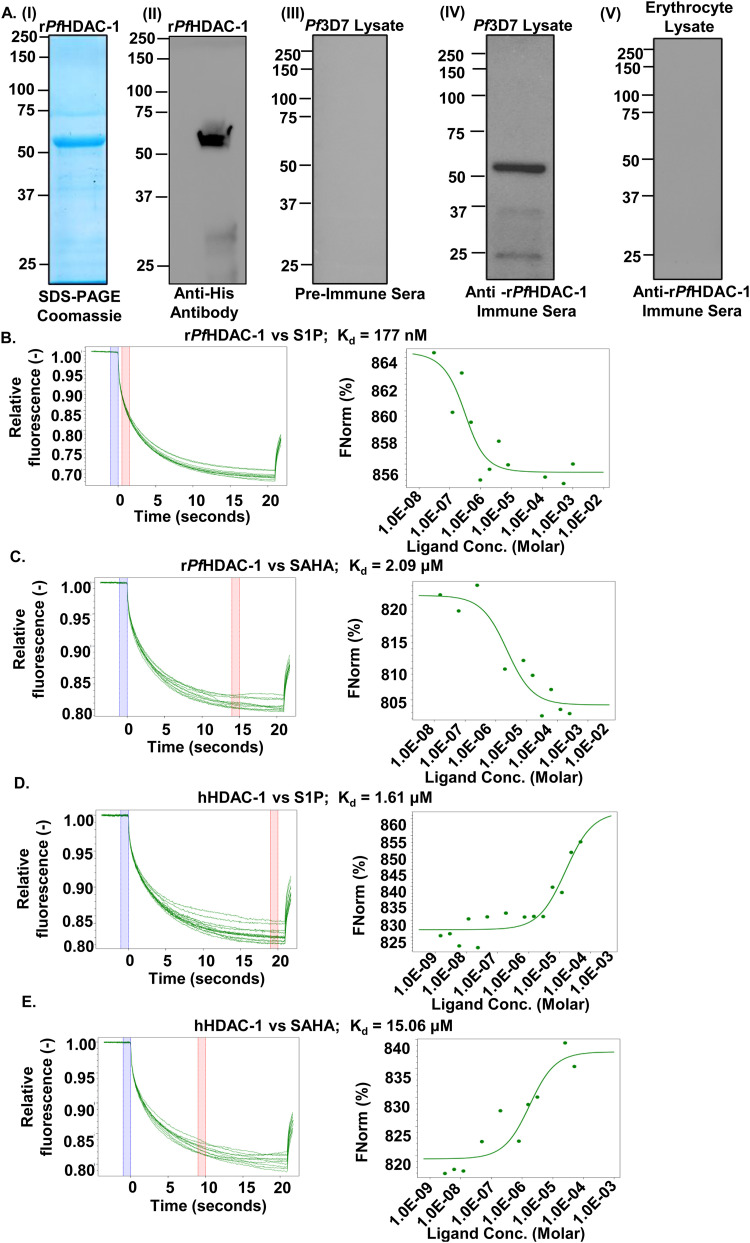
S1P interacts with r*Pf*HDAC-1. (A, panel I) Coomassie-stained polyacrylamide gel showing affinity purified r*Pf*HDAC-1 with an expected size of ~52 kDa. (A, panel II) Immunoblot detection of r*Pf*HDAC-1. Immunoblot analysis of: pre-immune mouse sera showing no reactivity with *Pf*3D7 proteins (A, panel III); in-house generated polyclonal mouse sera confirming the expression of *Pf*HDAC-1 (~52 kDa) by the parasite (A, panel IV); r*Pf*HDAC-1 antiserum showing no reactivity with the erythrocyte lysate proteins (A, panel V). (B to E). MST analysis confirms the interaction between HDAC-1 and S1P. MST traces (left) and dose-response curves (right) of r*Pf*HDAC-1-S1P (K_d_: 177 nM) (B), r*Pf*HDAC-1-SAHA (K_d_: 2.09 μM) (C), hHDAC-1-S1P (K_d_: 1.61 μM) (D), and hHDAC-1-SAHA (K_d_: 15.06 μM) (E). The known HDAC binder, SAHA was used as a positive control.

In humans, the pleiotropic lipid mediator, S1P has been shown to regulate histone acetylation by specifically binding to and inhibiting the enzymatic activities of histone deacetylases: HDAC-1 and HDAC-2, which prevents the removal of acetyl groups from lysine residues in histone tails. This demonstrated the connecting link between nuclear S1P and epigenetic regulation of gene expression via HDACs, which are direct intracellular targets of S1P (15). In the malaria parasite P. falciparum, *Pf*HDAC-1 has recently been shown to serve as one of the epigenetic modifiers of the histone acetylation code, which upon phosphorylation, binds to and regulates crucial genes associated with both housekeeping (entry into the host cell, hemoglobin metabolism, and cell cycle) and stress-responsive (protein folding and redox homeostasis) functions, thus acting as a key regulator of the gene expression profile of the parasite (13). However, S1P-mediated regulation of *Pf*HDAC-1 is not completely understood. Towards this, we overexpressed and purified 6×-His-*Pf*HDAC-1 (Fig. 1A, panels I and II, and Fig. S1), followed by the generation of anti-*Pf*HDAC-1 immune sera and validation of its specificity against recombinant *Pf*HDAC-1 (r*Pf*HDAC-1) protein using immunoblotting (Fig. 1A, panels III and IV). The antiserum showed no reactivity with the erythrocyte lysate proteins (Fig. 1A, panel V). *In vitro* interaction of S1P with r*Pf*HDAC-1 and hHDAC-1 was monitored and quantified using Microscale Thermophoresis (MST), which is a novel method for immobilization-free interaction analysis (22, 23). When fluorescently labeled r*Pf*HDAC-1 and hHDAC-1 were titrated against S1P, their thermophoretic mobility markedly changed, indicating their effective interaction with S1P. Calculation of equilibrium dissociation constants (K_d_) revealed K_d_ values of 177 ± 24 nM for S1P-r*Pf*HDAC-1, and 1.61 ± 0.8 μM for S1P-hHDAC-1, indicative of strong and specific interaction between S1P and *Pf*HDAC-1 (Fig. 1B and D). Suberoylanilide Hydroxamic Acid (SAHA), an HDAC inhibitor approved by the U.S. Food and Drug Administration for the treatment of cutaneous T-cell lymphoma, was taken as a positive control, which showed K_d_ values of 2.09 ± 0.45 μM for SAHA-*Pf*HDAC-1 and 15.06 ± 0.75 μM for SAHA-hHDAC-1 (Fig. 1C and E). Regarding the directionality of the dose-response curves displayed in Fig. 1B to E, we wish to underline that the amplitude of the response might be either negative or positive, depending on the precise influence of ligand binding on the target MST signal. The response amplitude will be negative if the complex’s MST signal (r*Pf*HDAC-1 + S1P/SAHA) (Fig. 1B and C) is lower than the target alone (labeled r*Pf*HDAC-1), and positive if the complex’s MST signal (hHDAC-1 + S1P/SAHA) (Fig. 1D and E) is higher than the target alone (labeled hHDAC-1). As also supported by the literature survey, both directions are frequent, and the direction of the response amplitude is irrelevant for interaction evaluation (24–29). Altogether, these results indicate that S1P could be an endogenous ligand of *Pf*HDAC-1.

### S1P interacts with, and regulates, *Pf*HDAC-1 enzymatic activity.

**FIG 2:**
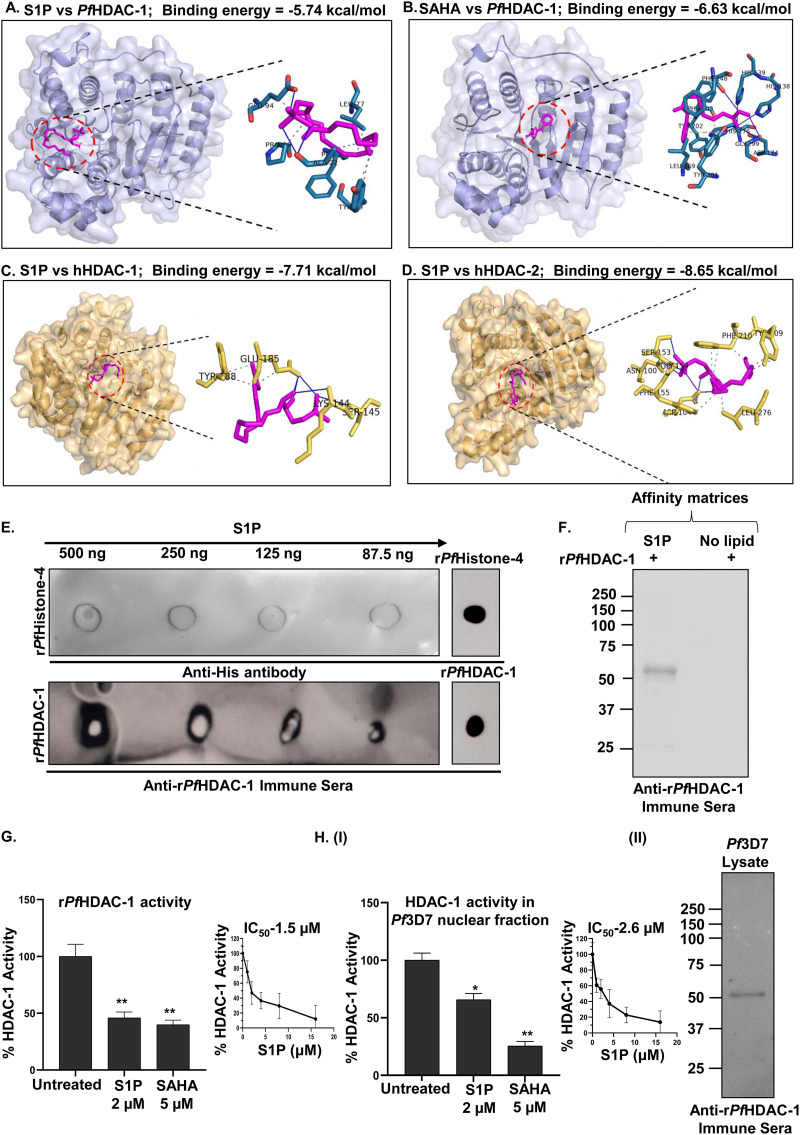
S1P modulates *Pf*HDAC-1 activity. Schematic representation of the interaction between S1P-*Pf*HDAC-1 (*ΔG*_bind_: −5.74 kcal/mol) (A), SAHA-*Pf*HDAC-1 (*ΔG*_bind_: −6.63 kcal/mol) (B), S1P-hHDAC-1 (*ΔG*_bind_: −7.71 kcal/mol) (C), and S1P-hHDAC-2 (*ΔG*_bind_: −8.65 kcal/mol) (D). (E) Protein-lipid overlay assay showing the interaction between S1P and r*Pf*HDAC-1. No significant binding was observed between S1P and r*Pf*Histone-4 (negative control). Spots of r*Pf*H4 and r*Pf*HDAC-1 were also applied to confirm the reactivity of the respective antibodies. (F) Pull-down of r*Pf*HDAC-1 using S1P-conjugated agarose beads, followed by immunoblotting. r*Pf*HDAC-1 pulled down with the control beads was used as a negative control. Effect of S1P (2 μM) on the enzymatic activity of r*Pf*HDAC-1 (G) and, native *Pf*HDAC-1 (H, panel I) from the parasite’s nuclear fraction. A significant reduction (~50%) in the enzymatic activity was observed upon treatment with S1P, which was comparable to the treatment with SAHA (5 μM). Insets represent the percent change in the enzymatic activities in response to increasing S1P concentrations (*n* = 3; Student's *t* test; *, *P ≤ *0.05). (H, panel II) Immunoblot showing the presence of native *Pf*HDAC-1 in the parasite’s nuclear fraction which was used for the enzymatic assays.

We examined the binding properties of S1P and *Pf*HDAC-1 using an *in silico* interaction analysis approach, wherein, docked conformations with the highest (most negative) binding energy (*ΔG*_bind_) were selected and analyzed. S1P showed *ΔG*_bind_ of −5.74 kcal/mol, forming H-bonds (with ALA22 and GLU94), and hydrophobic interactions (with 7 residues) with *Pf*HDAC-1 (Fig. 2A, Fig. S2, and Table S2). Likewise, SAHA (positive control) showed *ΔG*_bind_ of −6.63 kcal/mol, forming several close interactions such as H-bonds (with HIS139 at the active site, GLY147 at the substrate-binding site, ASP174, HIS176 at the metal ion-binding site, and TYR301) and hydrophobic interactions (with TYR301 at the substrate-binding site) offered by *Pf*HDAC-1 (Fig. 2B and Table S5). Furthermore, we docked S1P with hHDAC-1 and hHDAC-2 proteins. We found that S1P interacts with hHDAC-1 via 3 hydrophobic contacts and 5 H-bond interactions, with *ΔG*_bind_ of −7.71 kcal/mol (Fig. 2C and Table S3). Similarly, we found that S1P interacts with hHDAC-2 via 7 hydrophobic contacts and H-bond interactions (Table S4), with *ΔG*_bind_ of −8.65 kcal/mol (Fig. 2D). These findings suggested that S1P forms energetically stable complexes with the HDAC proteins. Additionally, hHDAC-1 and hHDAC-2 revealed a 60% and 27 to 35% sequence identity with *Pf*HDAC-1 and *Pf*HDAC-2, respectively, as shown in Fig. S3 and 4.

The interaction between *Pf*HDAC-1 and S1P was confirmed *in vitro* by a protein-lipid overlay assay (30), in which the dot blot intensity scale of r*Pf*HDAC-1 directly correlated with S1P in a dose-dependent manner, indicating the direct interaction between the two (Fig. 2E). No such signal was observed when S1P was incubated with r*Pf*Histone 4 (r*Pf*H4). To confirm the interaction between S1P and *Pf*HDAC-1, we performed a pull-down assay using S1P-conjugated agarose beads and r*Pf*HDAC-1. Notably, a distinct band of r*Pf*HDAC-1 was observed with an apparent molecular mass of ~52 kDa (Fig. 2F). No such band was observed when control unconjugated agarose beads were used.

To determine if the interaction with S1P has any impact on the enzymatic activity of *Pf*HDAC-1, we performed a fluorescence-based HDAC activity assay as mentioned in the material and methods section. Interaction with S1P reduced r*Pf*HDAC-1 activity by 50%, as indicated by the difference in fluorescence intensity between untreated and S1P-treated samples (Fig. 2G). An enzymatic reaction in the presence of SAHA was taken as a positive control. Similar results were obtained using endogenous *Pf*HDAC-1 from the parasite’s nuclear fraction (Fig. 2H, panel I). Moreover, immunoblot analysis with anti-r*Pf*HDAC-1 immune sera confirmed the presence of native *Pf*HDAC-1 in the parasite lysate (Fig. 2H, pane lII). These findings strongly suggest that S1P binds to *Pf*HDAC-1 and inhibits its enzymatic activity *in vitro*.

### Inhibition of host SphK-1 reduces S1P levels in the parasite.

**FIG 3:**
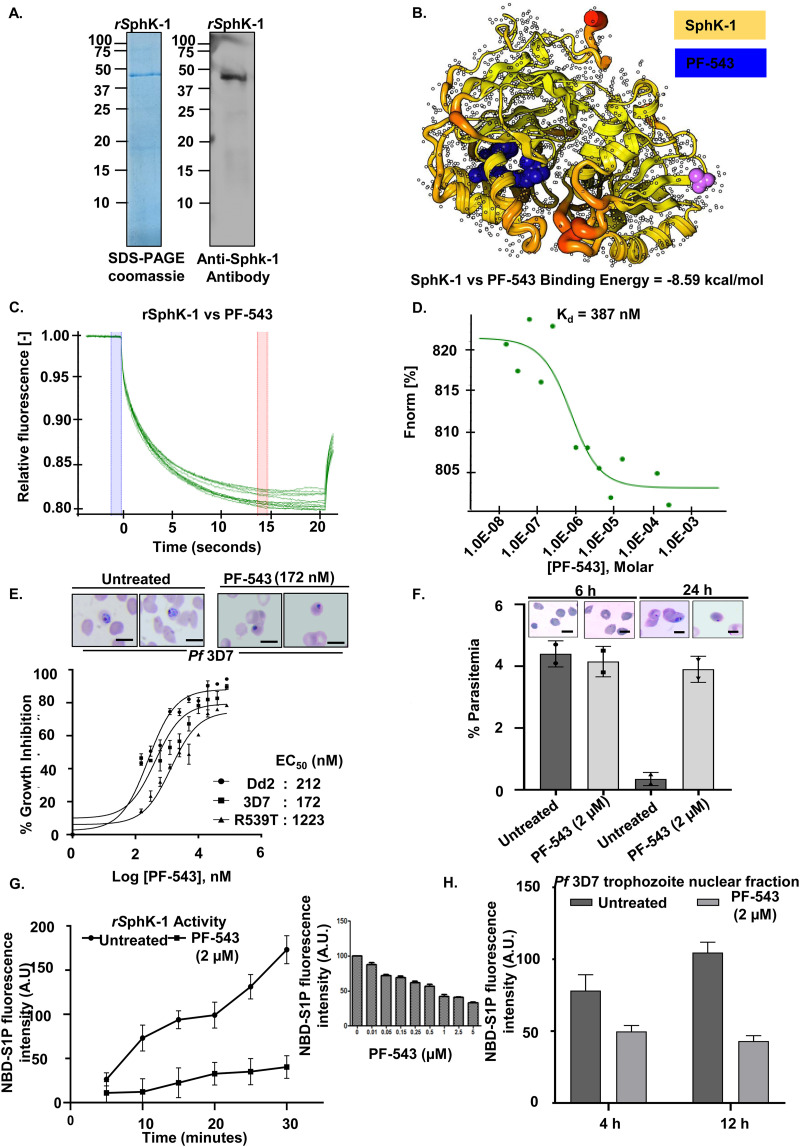
PF-543 inhibits hSphK-1 activity. (A) Coomassie-stained polyacrylamide gel showing affinity purified rSphK-1 with an expected size of ~45 kDa, which was validated by immunoblotting. (B) Schematic representation of complex formation between PF-543 and SphK-1 (*ΔG*_bind_: −8.59 kcal/mol). (C and D). MST analysis confirmed the interaction of PF-543 with rSphK-1. MST traces (C) and dose-response curve (D) show a K_d_ value of 387 nM. (E) *In vitro* growth-inhibition curves of *Pf*3D7, *Pf*Dd2, and *Pf*R539T parasites upon treatment with PF-543 for 72 h yielded EC_50_ values of 172 nM (*Pf*3D7), 212 nM (*Pf*Dd2), and 1223 nM (*Pf*R539T) (*n* = 3; non-linear regression analysis). (F) Uninfected erythrocytes were treated with PF-543 (2 μM) for 2 h, parasitized with the purified *Pf*3D7 schizonts, and Giemsa-stained smears were prepared 6 and 24 h postinfection. No significant difference in the merozoite invasion was observed between untreated and treated samples 6 h postinfection. After 24 h of infection, the number of dead parasites was higher in the treated sample compared to the untreated one. The representative Giemsa-stained images for each condition has been shown in the inset (*n* = 3; Student's *t* test); scale bar: 5 μm. (G) NBD-S1P-based fluorometric assay showing PF-543 (2 μM)-mediated inhibition of rSphK-1 activity (*n* = 3). The inset shows enzymatic activity of rSphK1 in the presence of different concentrations of PF-543, which revealed the IC_50_ value of PF-543 to be 2 μM. (H) Bar graph depicting S1P levels in *Pf*3D7 trophozoite’s nuclear fraction, at time intervals of 4 h and 12 h, upon PF-543 (2 μM)-mediated inhibition of SphK-1 (*n* = 3; Student's *t* test; *, *P ≤ *0.05).

Literature review suggests that sphingolipids serve crucial functions in regulating microbial pathogenesis. For instance, some pathogens, such as bacteria and viruses, exploit host sphingolipids to promote development and infection, whereas fungus-host interactions involve both host and fungal sphingolipids (31–33). As a result, we assessed the level of S1P in P. falciparum-infected erythrocytes in the presence of PF-543, a potent, selective, reversible, and sphingosine-competitive inhibitor of SphK1-catalyzed sphingosine phosphorylation (34). For this purpose, we purified 6×-His-hSphK-1 (Fig. 3A), and examined its binding affinity and enzymatic activity in the presence of PF-543. *In silico* interaction analysis showed that PF-543 interacts with hSphK-1 with *ΔG*_bind_ of −8.59 kcal/mol (Fig. 3B and Fig. S5). The interaction of PF-543 with hSphK-1 was monitored and quantified using MST, wherein, fluorescently labeled hSphK-1 was titrated with PF-543. Thermophoretic mobility of hSphK-1 markedly changed, indicating an effective interaction with PF-543, with a K_d_ value of 387 nM (Fig. 3C and D, and Fig. S5). Thus, molecular docking studies and MST analysis clearly demonstrated a significant interaction between hSphK-1 and PF-543.

To decipher the relationship between host SphK-1 activity and parasite growth, PF-543 was evaluated *in vitro* for antimalarial activity against intra-erythrocytic stages of P. falciparum strain 3D7 (chloroquine and artemisinin-sensitive). It is imperative that to validate therapeutic usefulness, new antimalarial medications undergo cross-resistance studies to ensure that they withstand current parasite resistance mechanisms. Therefore, PF-543 was also evaluated for antimalarial activity against P. falciparum strains Dd2 (chloroquine-resistant) and R539T (artemisinin-resistant). The results showed that PF-543 significantly reduced parasite load, with EC_50_ values of 212 nM (Dd2), 172 nM (3D7), and 1223 nM (R539T) (Fig. 3E). We also examined the effect of PF-543 (2 μM) treatment on the invasion rate of the *Pf*3D7 parasites. It was observed that even after 6 h of treatment, the percent parasitemia of untreated and treated samples remained the same, suggesting that the invasion rate was not affected by PF-543. However, 24 h post-treatment, dead parasites were observed in the PF-543 treated sample compared to the untreated sample (Fig. 3F). These findings support the hypothesis that treatment with PF-543 can impair *de novo* synthesis of S1P in erythrocytes. As a result, in the case of infected erythrocytes, the parasite will no longer be able to utilize the host S1P pool, resulting in attenuated growth.

**FIG 4:**
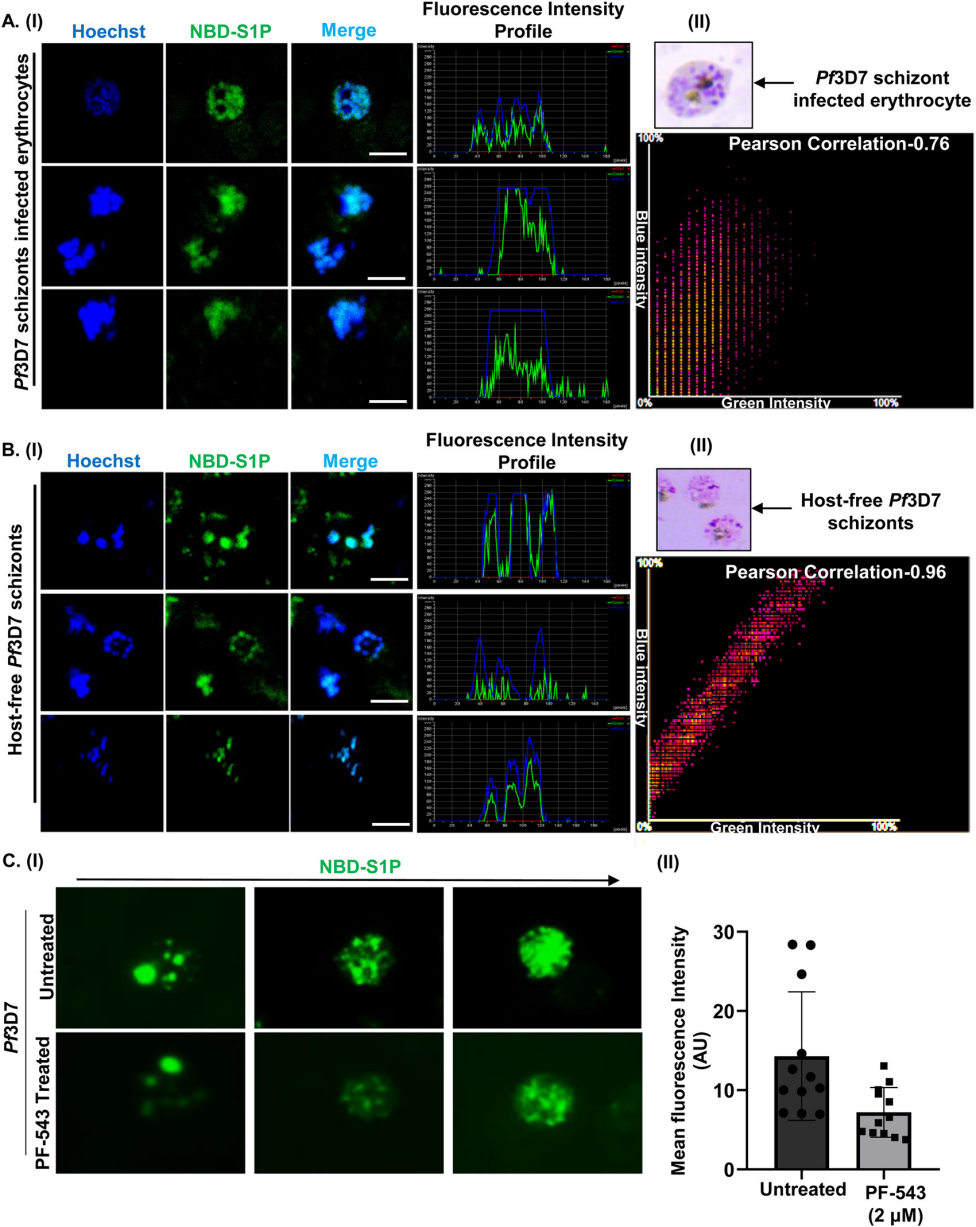
Uptake of host S1P by the parasite. (A, panel I) Fluorescence microscopic images showing the uptake of NBD-Sph by *Pf*3D7 schizont-infected erythrocytes. The presence of NBD-S1P (green) in the parasite nuclei is depicted by its co-localization with Hoechst nuclear stain (blue). Scale bar: 5 μm. (A, panel II) Co-localization is further depicted by a representative scatterplot in which the green channel (NBD-S1P) is shown on the *x* axis and the blue channel (Hoechst) is shown on the *y* axis (Pearson’s correlation coefficient: 0.76). The inset shows a representative Giemsa-stained image of *Pf*3D7 schizont-infected erythrocyte. (B, panel I) Fluorescence microscopic images depicting the NBD-S1P levels in the host-free *Pf*3D7 parasites. NBD-S1P (green) was found to localize in the parasite nuclei (Hoechst; blue). Scale bar: 5 μm. (B, panel II) A representative scatterplot depicting co-localization, in which the green channel (NBD-S1P) is shown on the *x* axis, and the blue channel (Hoechst) is shown on the *y* axis (Pearson’s correlation coefficient: 0.96). The inset shows a representative Giemsa-stained image of the host-free *Pf*3D7 schizont. (C, panel I) Fluorescence micrographs indicating a reduction in the fluorescence intensities of NBD-S1P (green) in the parasite nucleus upon PF-543 (2 μM) treatment. (C, panel II) Estimates of mean fluorescence intensities of NBD-S1P in each condition are shown as a bar graph (*n* = 3; Student's *t* test; *, *P ≤ *0.05).

To further investigate the effect of PF-543 on the catalytic activity of hSphK-1, we performed a fluorimetry-based enzymatic assay using NBD-sphingosine, a derivative of sphingosine tagged with the fluorophore, 7-nitro-2-1,3-benzoxadiazol-4-yl (NBD) group, and examined the formation of NBD-S1P from NBD-sphingosine (35). Interestingly, PF-543 treatment resulted in a time-dependent reduction in NBD-S1P levels, as done using both rSphK-1 (Fig. 3G) and the parasite’s nuclear fraction (Fig. 3H), in contrast to the untreated control. The enzymatic activity of rSphK1 in the presence of different concentrations of PF-543 revealed an IC_50_ value of 2 μM (inset of Fig. 3G). Furthermore, the viability of the parasites was not compromised, even after 4 and 12 h of PF-543 treatment (Fig. S7), which validates that the reduction of S1P signals in the parasite nucleus after treatment with PF-543 for the specified time intervals was not because the parasites were dead after treatment. The reduction in NBD-S1P levels was corroborated by fluorescence imaging of its intracellular localization in infected erythrocytes and saponin-lysed parasites. Fluorescence micrographs and line graph analysis clearly demonstrated the uptake of NBD-S1P and its co-localization with Hoechst nuclear stain in infected erythrocytes (Fig. 4A and Fig. S8) and host-free parasites (Fig. 4B). Concomitantly, we observed gradual decline in the S1P levels in the RBC cytosol as the parasite progressed from ring to schizont stage. This is consistent with our previous finding (Sah et al., 2020), in which we demonstrated that during the intra-erythrocytic proliferation cycle of the parasite, following 10 to 42 h post-invasion (hpi), the host SphK-1 levels and its functional activity (and thus, the intra-erythrocytic S1P levels) are gradually suppressed (by an unknown mechanism) as the parasite progresses from ring to schizont stage, that directly correlates with the reduced S1P production *in vitro* (Sah et al., 2020) (19). Furthermore, parasites isolated from infected erythrocytes after 30 min of incubation with NBD-Sphingosine displayed considerable NBD-S1P uptake, which reduced to 50% after treatment with PF-543, indicating the parasite’s codependency on the host S1P pool (Fig. 4C, panel I). The observed difference in NBD-S1P fluorescence intensity was reflected in the mean fluorescence intensity bar graph (Fig. 4C, panel II). In summary, these findings show that host S1P localizes to parasite nuclei, and that any modulation in S1P synthesis in the infected erythrocytes reduces parasite S1P uptake.

### Host S1P alters histone acetylation in parasite nuclei via interacting with *Pf*HDAC-1.

**FIG 5:**
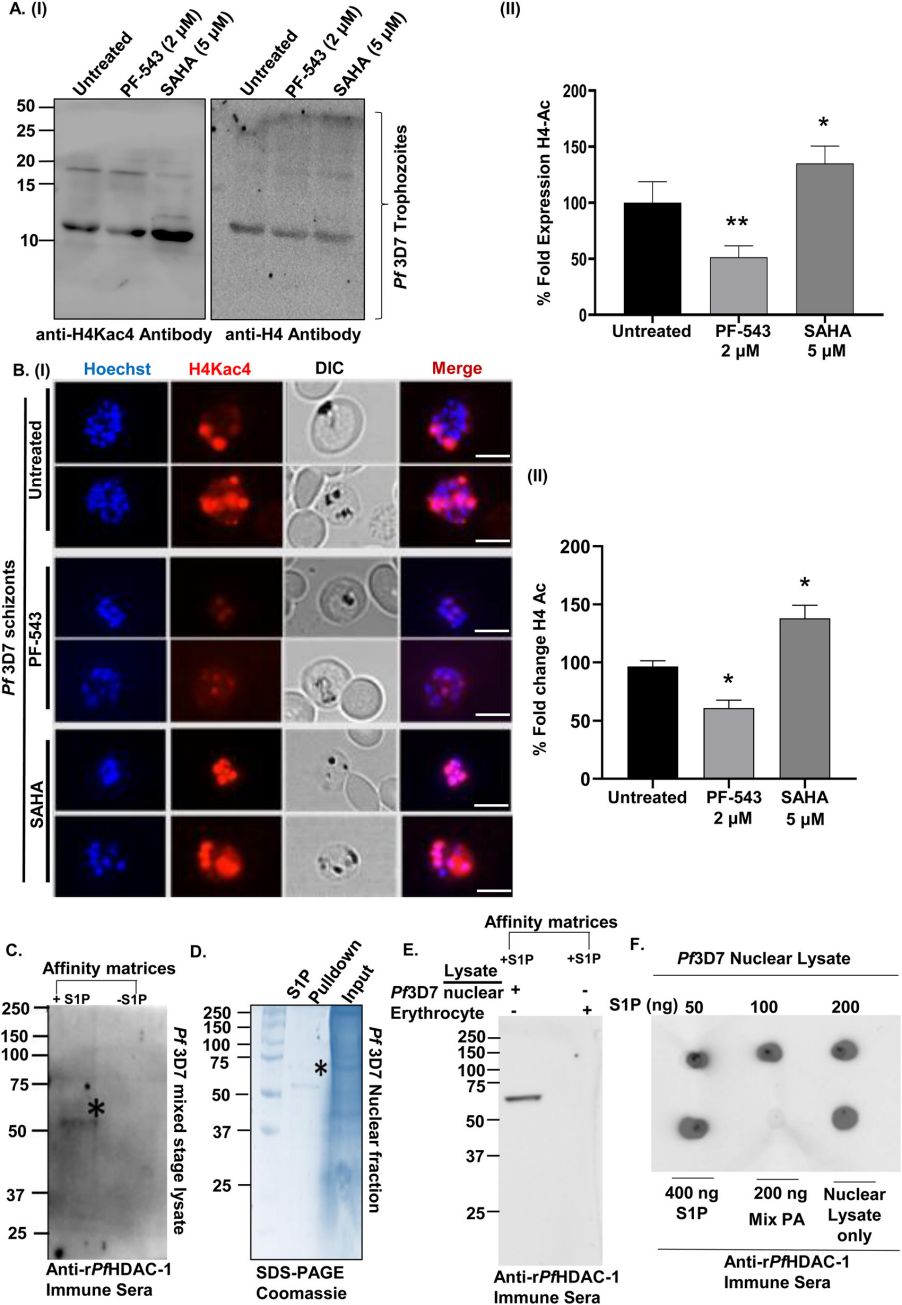
Host S1P regulates *Pf*HDAC-1-mediated deacetylation of histone. (A, panel I) Immunoblot showing the relative histone acetylation (H4Kac4) status in the nuclear extracts of *Pf*3D7 trophozoites upon treatment with DMSO (0.005%), PF-543 (2 μM), and SAHA (5 μM). Histone-H4 served as a loading control. (A, panel II) Bar graph depicting quantification of the histone acetylation status for each experimental condition, with respect to H4 (*n* = 5; Student's *t* test; *, *P < *0.05; **, *P* < 0.005). (B, panel I) Immunofluorescence micrographs of erythrocytes parasitized with *Pf*3D7 schizonts showing reduced histone acetylation upon treatment with PF-543 (2 μM) and SAHA (5 μM). The parasite nuclei are stained with Hoechst (blue). Scale bar: 5 μm. (B, panel II) Bar graph representing the percent fold change of H4Kac4 levels from each condition (*n* = 3; Student's *t* test; *, *P < *0.05). (C) Immunoblot analysis of the native *Pf*HDAC-1 pulled down from *Pf*3D7 mixed stage (ring, trophozoite, and schizont) lysate using S1P-conjugated agarose beads. (D) Coomassie-stained polyacrylamide gel showing a prominent band of native *Pf*HDAC-1 (D, lane 1) obtained from the pulldown assay. (D, lane 2) The crude nuclear lysate (input) used in the pulldown assay. (E) Immunoblot depicting the presence of *Pf*HDAC-1 in the parasite’s nuclear fraction upon pull-down with S1P-conjugated beads. HDAC-1 could not be detected in the erythrocyte lysate. (F) Protein-lipid overlay analysis showing the interaction of S1P with native *Pf*HDAC-1 from *Pf*3D7 nuclear fraction. No binding was observed with phosphatidic acid (negative control). The crude nuclear fraction of the parasite lysate was used as a positive control.

It is well established that DNA replication requires chromatin decompaction, which is mediated by histone acetylation. As a result, we examined the effect of S1P levels (altered with PF-543) and HDAC inhibitor, SAHA, on *Pf*Histone-4 acetylation using immunoblotting. In comparison to the untreated control, the acetylation signals obtained after probing with anti-(tetra)-acetyl histone H4 (H4Kac) antibodies were significantly decreased after treatment with PF-543, and increased after treatment with SAHA (Fig. 5A, panels I and II). *Pf*H4 was used as a loading control. *Pf*H4 acetylation signals in the presence of PF-543 and SAHA were confirmed by fluorescence imaging of histone acetylation in the late trophozoite and schizont stages of *Pf*3D7 parasites. Hoechst staining and *Pf*H4Kac immunolabeling identified histone acetylation in the parasite nuclei, which decreased upon treatment with PF-543 and increased upon SAHA treatment (Fig. 5B, panel I), as depicted in the form of a histogram (Fig. 5B, panel II).

Our preliminary findings on the interaction of host erythrocyte-derived S1P and r*Pf*HDAC-1 prompted us to establish the same in the parasite. Pull-down of *Pf*HDAC-1 from the parasite lysate using S1P-conjugated agarose beads, followed by immunoblotting with anti-r*Pf*HDAC-1 antibody, verified the interaction (Fig. 5C). Additionally, a pull-down assay was performed with the parasite’s nuclear lysate and erythrocytes, followed by immunoblotting with anti-r*Pf*HDAC-1 antibody. A band corresponding to ~52kDa in the nuclear lysate, but not in the erythrocytes, demonstrated that S1P interacts only with *Pf*HDAC-1 and not hHDAC-1 (Fig. 5D and E). The interaction was validated by a protein-lipid overlay assay (30), in which the dot blot intensity scale of *Pf*HDAC-1 correlated with S1P in a dose-dependent manner, demonstrating a direct interaction between the two (Fig. 5F). The absence of any signal with Phosphatidic Acid (PA) showed selectivity of *Pf*HDAC-1 for S1P. These findings indicate that S1P interacts with *Pf*HDAC-1 in the parasite nuclei, and modulates the enzyme activity.

### Downregulation of host S1P alters *Pf*HDAC-1 mediated gene expression.

**FIG 6:**
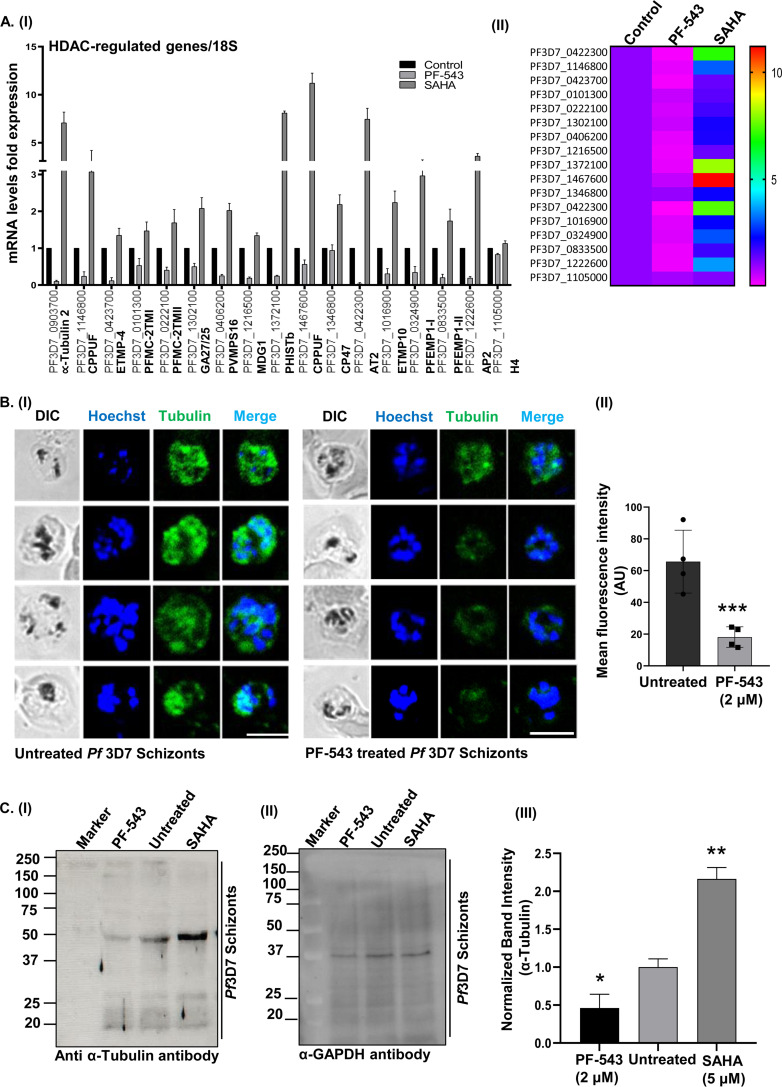
S1P-mediated *Pf*HDAC-1 regulation alters the overall transcriptional response of P. falciparum. (A, panel I) qRT-PCR analysis of 17 parasite genes, out of which 8 genes were found to be upregulated and downregulated (in both biological replicates) upon treatment of the parasite with SAHA (5 μM) and PF-543 (2 μM), respectively. Relative fold change in the gene expression levels was calculated using the 2^−ΔΔCt^ method, where 18S rRNA was used as an internal control. (A, panel II) A representative heat map showing the transcriptional changes of the *Pf*HDAC-1-regulated genes, upon treatment with SAHA and PF-543. (B, panel I) Immunofluorescence analysis to show alteration in α-tubulin expression upon treatment of *Pf*3D7 schizonts parasitized erythrocytes with PF-543 (2 μM). The parasite nuclei were stained with Hoechst (blue). Scale bar: 5 μm. (B, panel II) Bar graph depicting the average fluorescence intensity for each condition (*n* = 3; Student's *t* test; *, *P < *0.05; ***, *P* < 0.0005). (C, panel I) Immunoblot analysis to validate the relative expression of α-tubulin in the parasites treated with PF-543 (2 μM) and SAHA (5 μM). (II) GAPDH (~38 kDa) was used as a loading control. (C, panel III) Bar graph summarizing the average intensity of α-tubulin for each condition, normalized with GAPDH (*n* = 3; Student's *t* test; *, *P < *0.05; **, *P* < 0.005).

According to the literature, small molecule-mediated HDAC inhibition results in genome-wide transcriptional alterations in P. falciparum (21). *Pf*HDAC-2 has been linked to parasite transmissibility and virulence by functioning as a global suppressor of virulence gene expression, and modulating the frequency with which the parasite switches from the asexual cycle to sexual development (36). Further, given that *Pf*HDAC-1 regulates several crucial biological processes associated with the parasite development and infection progression, including host cell invasion/egress, cellular signaling, hemoglobin metabolism, and cell cycle (13), we decided to investigate the effects of host S1P on *Pf*HDAC-1 mediated gene regulation during intraerythrocytic development of the parasite. We used RT-PCR to evaluate the expression profile of parasite genes previously reported to be altered (upregulated) by *Pf*HDAC inhibition (21, 36), upon treatment with PF-543 and SAHA (positive control). These genes included: α-tubulin, CPPUF-1, ETMP-4, PFMC-2TM-I, PFMC-2TM-II, GA27/25, PVMPS16, MDG1, PHISTb, CPPUF-2, CP47, AT2, ETMP10, PFEMPI-I, PFEMPI-II, and AP2-G (Table S1). The histone H4 gene was used as an internal control (21, 36), and RT-PCR using RNA isolated from untreated infected erythrocytes served as a negative control. Inhibition of hSphK1 by PF-543 resulted in a significant decrease in the expression of *Pf*HDAC regulated genes: α-tubulin (~90%, *P* ≤ 0.01), CPPUF-1 (~76%, *P* ≤ 0.03), ETMP-4 (~88%, *P* ≤ 0.02), PFMC-2TM-I (~47%, *P* ≤ 0.05), PFMC-2TM-II (~60%, *P* ≤ 0.02), GA27/25 (~50%, *P* ≤ 0.05), PVMPS16 (~75%, *P* ≤ 0.005), MDG1(~80%, *P* ≤ 0.03), PHISTb (~75%, *P* ≤ 0.04), CPPUF-2 (~44%, *P* ≤ 0.16), CP47 (~10%, *P* ≤ 0.003), AT2 (~95%, *P* ≤ 0.04), ETMP10 (~70%, *P* ≤ 0.01), PFEMPI-I (~65%, *P* ≤ 0.17), PFEMPI-II (~80%, *P* ≤ 0.02), and AP2-G (~80%, *P* ≤ 0.007) (Fig. 6A, panel I). A heat map depicting the expression profile of the genes upon treatment of the parasite with PF-543 and SAHA is shown in Fig. 6A, panel II.

Previous studies have reported that α-tubulin expression is a potential transcriptional marker of HDAC-1 suppression in the malarial parasite (9, 21). This is supported by the fact that PF-543 treatment reduced the expression of α-tubulin gene by 90% (Fig. 6A). To corroborate the RT-PCR-based association of HDAC-1 inhibition with α-tubulin expression, we performed an immunofluorescence assay at the schizont stage of *Pf*3D7 parasites, in the presence of PF-543. We used in-house generated polyclonal sera raised in mice against rα-tubulin (37). Following PF-543 treatment, the fluorescence intensity of α-tubulin decreased, as depicted in the fluorescence images (Fig. 6B, panel I). The cellular localization of tubulin was consistent with our previous findings (37, 38). The fluorescence intensities were plotted as a bar graph by measuring 10 individual cells each from the PF-543-treated and untreated samples (Fig. 6B, panel II). Furthermore, immunoblot analysis of parasites treated with PF-543 (2 μM) and SAHA (5 μM; positive control) for 8 h showed a desired band of α-tubulin (~42 kDa), consistent with our prior findings (37), which considerably declined following treatment with PF-543 and increased after SAHA treatment (Fig. 6C, panel I). GAPDH gene was used as a loading control (Fig. 6C, panel II). A bar graph depicting the normalized α-tubulin band intensity is shown in Fig. 6C, panel (III). Altogether, these findings establish S1P as an endogenous regulator of *Pf*HDAC-1-mediated gene expression in the parasite.

### hSphK-1 inhibition impairs sexual development of the parasite.

**FIG 7:**
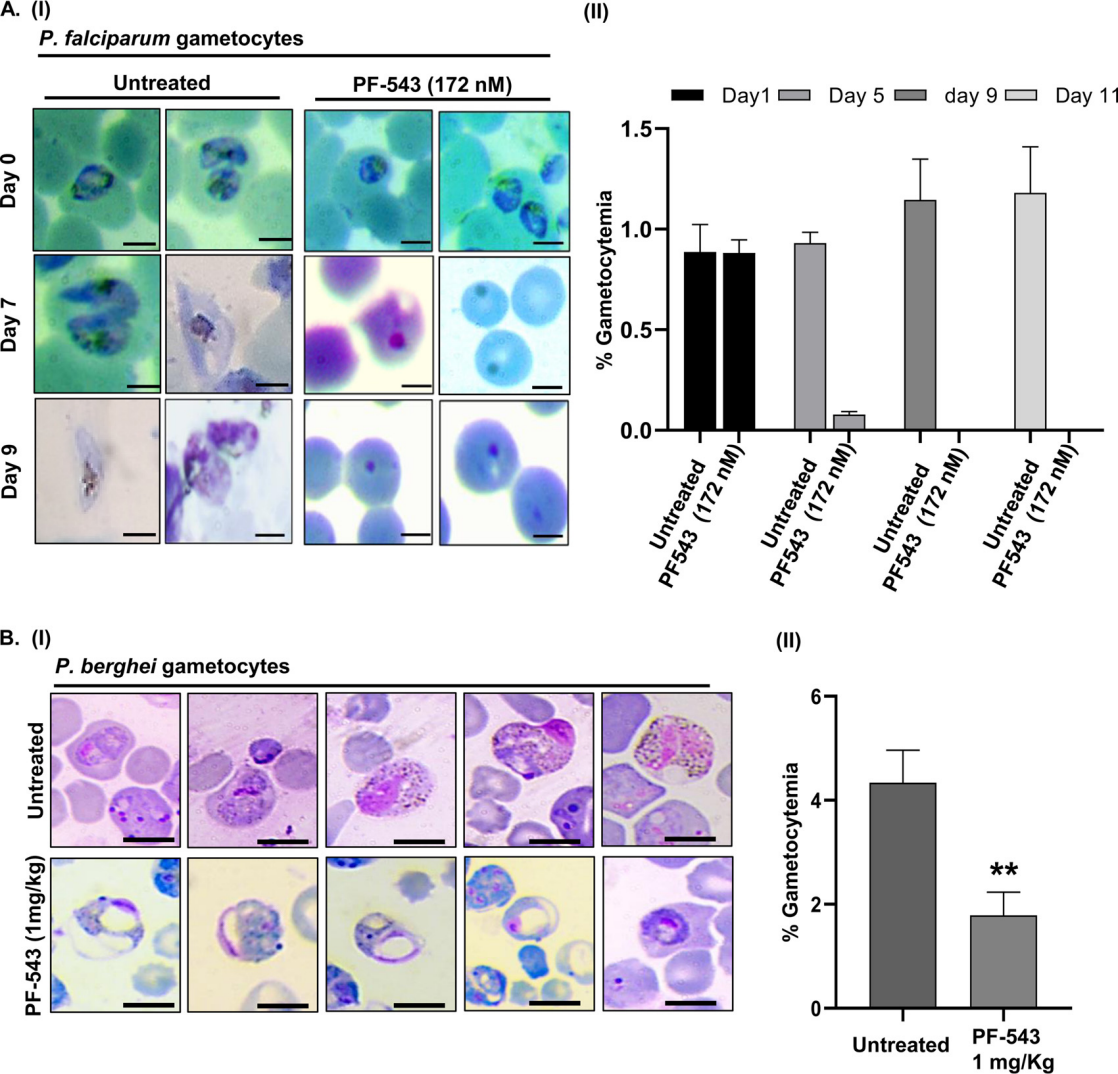
PF-543-mediated inhibition of *Pf*RKL-9 gametocyte maturation. (A, panel I) Giemsa-stained *Pf*RKL-9 parasites showing inhibition of gametocyte maturation upon treatment with PF-543 (172 nM), as indicated by the accumulation of pyknotic forms of the parasite 2 days post-treatment. Scale bar: 5 μm. (A, panel II). Bar graph depicting the average percent gametocytemia calculated for PF-543 (172 nM) treatment. Untreated *Pf*RKL-9 gametocytes were used as a negative control (*n* = 3; *, *P* < 0.05) (B, panel I) Giemsa-stained P. berghei ANKA parasites showing morphological deformities and a reduction in gametocytemia, following treatment with PF-543 (1 mg/kg). (B, panel II). Bar graph demonstrating a significant inhibition of gametocyte formation upon PF-543 (1 mg/kg) treatment (*n* = 3; *, *P* ≤ 0.05).

Expression profiles of the parasite genes (Fig. 6A and B) showed that *Pf*AP2-G, which is required for gametocyte maturation (39, 40), gets downregulated upon PF-543 treatment. *Pf*AP2-G has little effect on the asexual development of the parasite, but it is essential for sexual commitment and gametocyte maturation (39, 40). Evaluating the gametocidal activity of PF-543 in P. falciparum is, thus, necessary. *Pf*RKL-9 parasites were induced to differentiate into sexual stages before being treated with PF-543 at its EC_50_ of 172 nM, as derived from the intra-erythrocytic growth inhibition assay. A daily dose of PF-543 given to early stage I gametocytes halted the parasite growth, resulting in a decline in gametocytemia (Fig. 7A, panel I). By day 2, the majority of the parasites had developed pyknotic bodies. However, dimethyl sulfoxide (DMSO)-treated control parasites showed no growth retardation, and gametocytes developed till stage V. The observed difference in gametocytemia GAPDH gene was used as a loading control between the control and PF-543-treated parasites is depicted in the form of a histogram in Fig. 7A, panel II. Overall, these findings show that hSphk-1 inhibition attenuates early-stage gametocyte development.

### hSphK-1 inhibition impairs early sporogonic stages of P. berghei.

Lipid profiles of infected erythrocytes are typical of the parasite life cycle and gametocyte maturation. It has been reported that the cellular levels of phospholipids, which form a major class of membrane lipids, decline during gametocyte development. However, lipidomics analysis indicated an enrichment of sphingolipids and ceramides in gametocytes (41, 42). To assess the effect of the reduced host S1P levels on gametocytogenesis, mice infected with P. berghei and administered phenyl hydrazine were given PF-543 intraperitoneally. Representative Giemsa-stained smears showed healthy gametocytes in DMSO-treated mice. In contrast, immature and altered gametocytes were observed in PF-543-treated mice (Fig. 7B, panel I). The observed difference in gametocytemia between the vehicle-treated control and PF-543-treated mice is depicted as a histogram in Fig. 7B, panel II, which shows that gametocyte density significantly reduced by 50 to 60% 2 days post-treatment.

**FIG 8:**
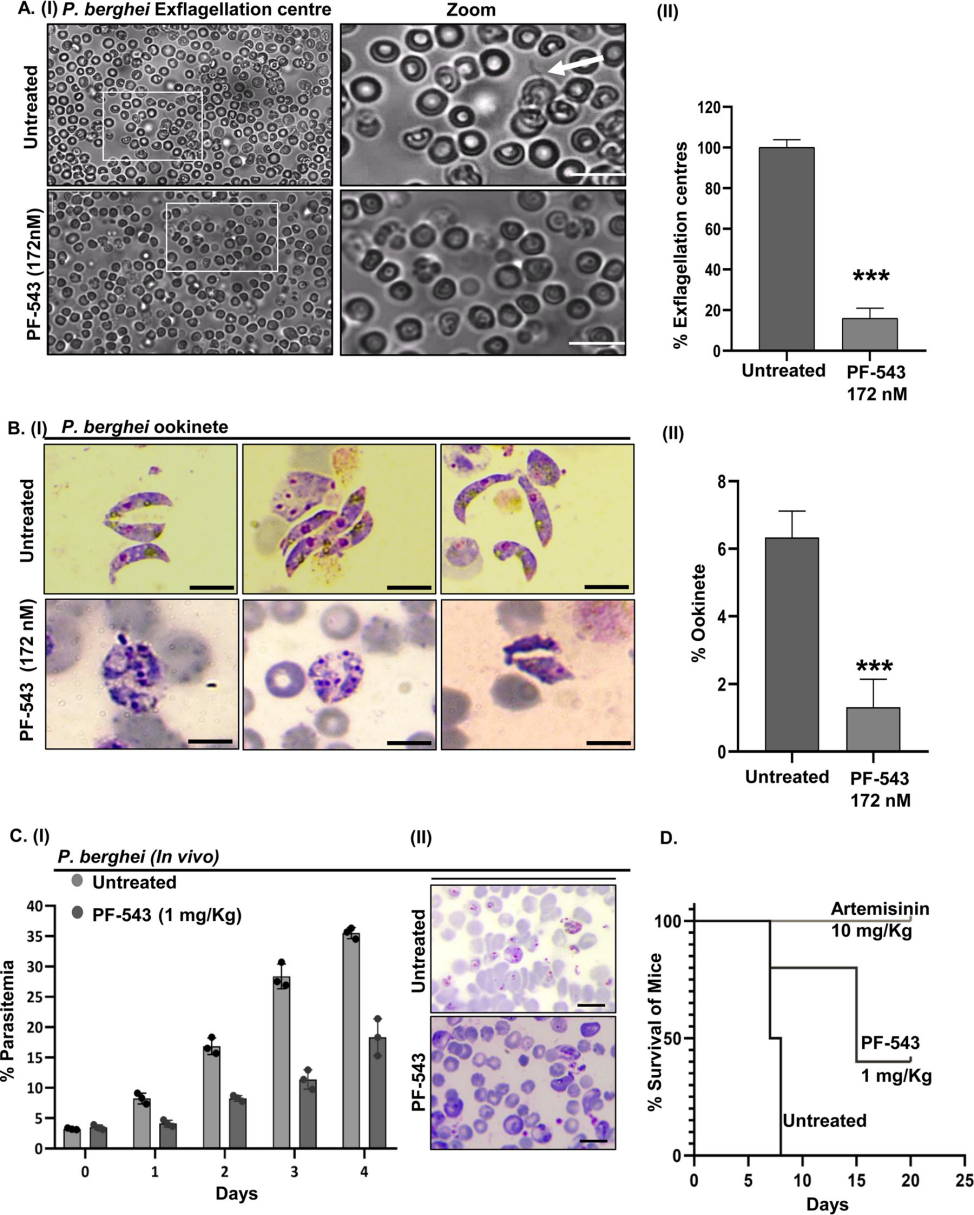
Effect of PF-543 treatment on the sporogonic cycle of P. berghei ANKA. (A, panel I) Light micrographs showing a drastic reduction in the exflagellation of *Pb* ANKA microgametocytes, upon treatment with PF-543 (172 nM). (A, panel II) Bar graph depicting percent exflagellation centers counted in 25 fields (*n* = 3; *, *P* < 0.05). (B, panel I) Giemsa-stained smears of *Pb* ANKA ookinetes showing morphological deformities and a reduction in ookinete formation upon PF-543 (172 nM) treatment. (B, panel II) Bar graph depicting a significant decline in ookinete formation by *Pb* ANKA parasites upon PF-543 (172 nM) treatment (*n* = 3; *, *P* < 0.05). (C, panel I) Growth inhibitory effect of PF-543 (1 mg/kg) on *Pb* ANKA confers enhanced mice survival, as indicated by a decline in parasitemia. (C, panel II) Giemsa-stained blood smears from untreated and PF-543 (1 mg/kg)-treated mice show a significant difference in parasitemia. (D) Survival plot demonstrating an increase in the mice survival upon administration with PF-543 (1 mg/kg). Mice treated with artemisinin (10 mg/kg) were taken as a positive control.

To evaluate the effect of PF-543 on male gamete exflagellation, we performed an *ex vivo* exflagellation assay with P. berghei infected RBCs. To differentiate between mobile male gametes and other stages of the parasite, the exflagellation centers were counted following DMSO and PF-543 treatment. After 15-20 min of ex-flagellation, male gametes emerged from the infected erythrocytes. Interestingly, PF-543-treated samples showed a significant reduction in ex-flagellation centers when compared to untreated control (Fig. 8A, panels I and II). This finding implicates the role of host S1P during male gamete exflagellation.

To anticipate suppression of parasite transmission, *in vitro* conversion of P. berghei gametocytes to ookinetes was evaluated following PF-543 treatment. At 24 h post-treatment, fully developed elongated ookinetes were observed in the DMSO-treated control group, whereas immature or partially developed ookinetes were observed in PF-543-treated group (Fig. 8B, panel I). Ookinete numbers were counted in each of the untreated and treated groups and represented in the form of a bar graph (Fig. 8B, panel II). PF-543 reduced ookinete formation by 80% compared to the untreated control, suggesting that ookinete maturation is mediated by host S1P. Epigenetic regulation of stage-specific *Plasmodium* gene expression is crucial. The aforementioned findings indicate that S1P modulates HDAC activity. Therefore, altering S1P levels will dysregulate the epigenetic regulation mediated by HDAC during the ookinete stage, thus impacting ookinete development as observed upon PF-543 treatment.

*In vitro* anti-plasmodial potency of PF-543 encouraged us to evaluate *in vivo* in mice infected with the murine malaria parasite, P. berghei strain ANKA. In contrast to the vehicle control, parasitemia was significantly reduced to 60% in PF-543 (1 mg/kg)-treated mice (Fig. 8C, panels I and II). Moreover, PF-543-treated mice outlived control mice by more than 12 days (Fig. 8D). Mice in the vehicle control group died within 7 days after infection. Furthermore, PF-543 treatment was well tolerated by all mice, as no significant side effects were observed. Artemisinin (10 mg/kg)-treated mice were taken as a positive control. These findings suggest that host SphK-1 inhibition can abolish intra-erythrocytic growth of P. berghei *in vivo*.

**FIG 9:**
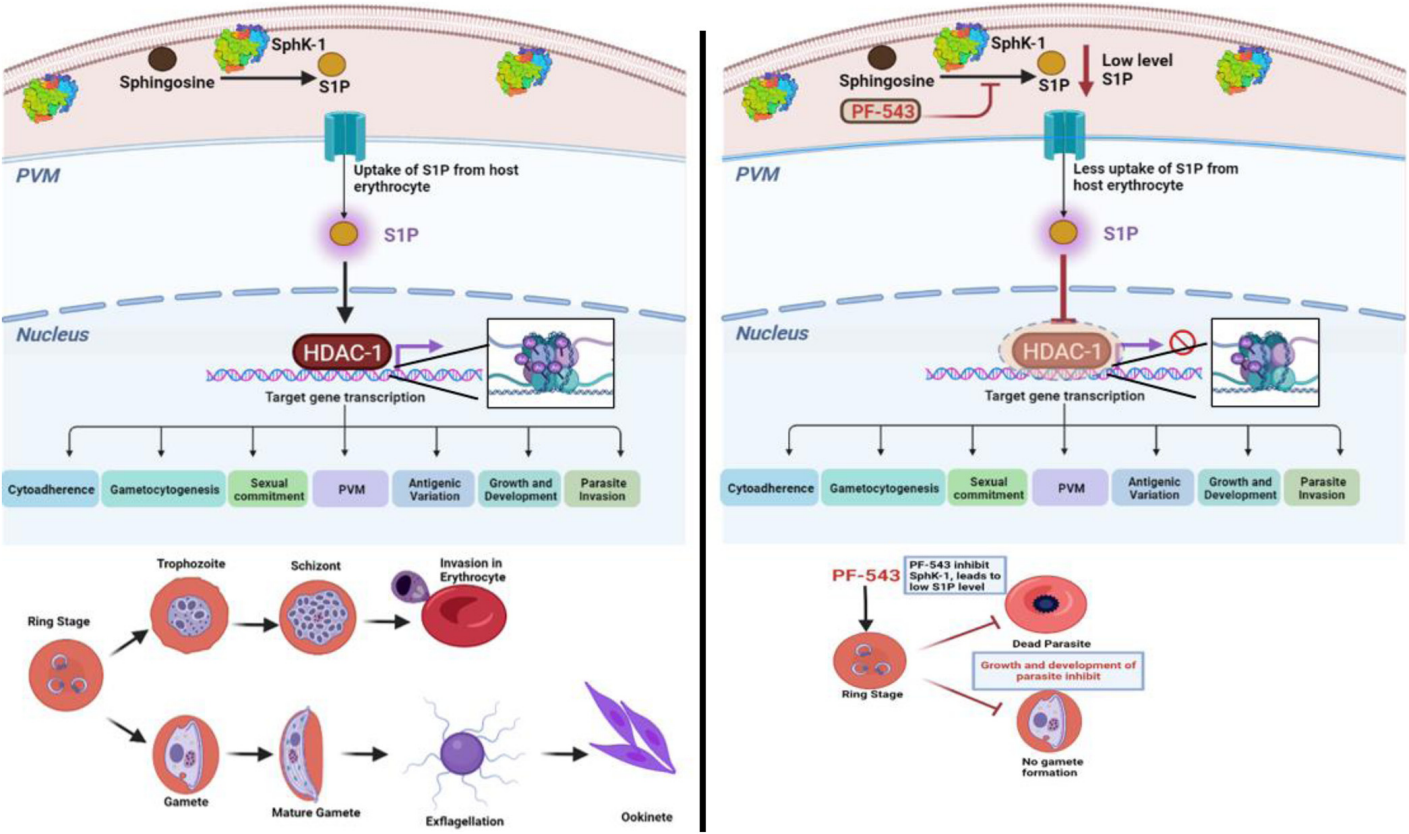
Schematic representation of S1P-mediated epigenetic modulation of parasite survival pathways (created with BioRender.com).

The model shown in Fig. 9 depicts the role of S1P as an endogenous regulator of *Pf*HDAC-1 activity. Sphk-1 is responsible for the synthesis of S1P inside the host. In the presence of its inhibitor, PF-543, S1P levels declines preventing S1P to interact with *Pf*HDAC-1, which results in the deacetylation of downstream genes associated with the *Plasmodium* life cycle, including asexual and sexual stage development.

## DISCUSSION

Sphingolipids are involved in several biological processes associated with microbial pathogenesis, including infection establishment and stimulation of host defenses against invading pathogens. Restricting the microbial use of sphingolipids has therefore been shown to reduce their pathogenicity (33). However, the functional role of sphingolipid metabolism and signaling in apicomplexan parasites, including *Plasmodium* species, is obscured.

Erythrocyte lipid rafts are associated with Glycosylphosphatidylinositol (GPI)-anchored, cytosolic, and transmembrane proteins, which get internalized in detergent-resistant membrane (DRM) fractions of the parasitophorous vacuolar membrane (PVM), suggesting that lipid rafts serve as a crucial platform for erythrocyte invasion of the parasite (43). It has been reported that sphingomyelin (SM) synthesis regulates the accumulation of DRM-associated proteins in the PVM (43). Various mammalian-*like* sphingolipid biosynthetic pathways have been identified in *Plasmodium*, which is attributed to sphingomyelin synthase (SMS) (18, 44–46) and glucosylceramide synthase (GCS) (17, 47). Specifically, SMS harbored within tubulovesicular membrane (TVM) serves as a vital interface between the intracellular parasite and the extracellular domain for the uptake of nutrients, and host proteins during parasite development (43, 48). Inhibiting SMS activity, in particular, has been shown to result in parasite mortality, implying that sphingomyelin synthesis is essential for *Plasmodium* survival and virulence. The genome of P. falciparum also encodes sphingomyelinase (SMase) (49, 50), which is hypothesized to metabolize host SM and generate ceramide, which is subsequently used by the plasmodial SMS and GCS to produce SM and glucosylceramide (GlcCer) in the TVM (32). Therefore, the literature suggests that the malaria parasite expresses orthologous genes involved in sphingolipid synthesis; however, the critical ones are missing.

P. falciparum uses both its *de novo* pool and the host cell lipid repertoire during intra-erythrocytic development and proliferation (20, 41, 51). Given the fact that the P. falciparum genome does not encode for SphK and S1P-degrading enzymes (lyases and phosphohydrolases), and acquires sphingosine from the environment, erythrocytes serve as the major reservoir of circulating S1P (52). We recently demonstrated parasite codependency on the endogenous S1P pool of host erythrocytes, and characterized the associated cellular mechanisms in the parasite (19, 20). However, it is critical to investigate if the host S1P serves as a signaling molecule and what its receptor in the parasite might be.

Nuclear S1P has been reported to alter histone acetylation states and confer epigenetic regulation of gene expression by selectively binding to, and inhibiting the functional activity of HDACs (15). However, the molecular mechanism by which S1P regulates *Pf*HDAC-1 is not completely understood. Here, we have used combinatorial approaches such as chemical, *in silico* analysis, biophysical and biochemical procedures (both *in vitro* and *ex vivo*), and showed that S1P selectively interacts with and inhibits the enzymatic activity of *Pf*HDAC-1 in the parasite nuclei.

To explore the regulation of *Pf*HDAC-1 by S1P, we intended to employ an experimental genetics approach. However, by knocking down hSphK-1 in immortalized adult erythroid line, Bristol Erythroid Line Adult (BEL-A), we were unable to generate terminally differentiated erythrocytes devoid of SphK-1. Suppression of SphK-1 has recently been shown to enhance erythroid cell apoptosis *in vitro* (53). Moreover, *Pf*HDAC-1 plays a crucial role in parasite development (13). As a result, knocking down SphK-1 in erythroid progenitor cells is not a suitable method for establishing the relevance of S1P-mediated signaling in the parasite. Therefore, using the SphK-1-specific inhibitor, PF-543, appeared to be the preferable approach for determining the importance of S1P signaling in the parasite epigenetic reprogramming (34, 54–57).

We assessed S1P levels in P. falciparum-infected erythrocytes following the suppression of its *de novo* synthesis by inhibiting hSphK-1 activity with PF-543. We showed a significant interaction between PF-543 and hSphK-1. Further, the inhibition of hSphK-1 resulted in attenuated intra-erythrocytic growth of P. falciparum strains 3D7, Dd2 (chloroquine-resistant), and R539T (artemisinin-resistant), with EC_50_ values in the nanomolar range. Interestingly, by modulating the host S1P synthesis with PF-543, a time-dependent reduction in S1P levels in *Pf*3D7-infected erythrocytes and host-free parasites was observed. These findings directly demonstrate “lipid sequestration” as a mechanism by which sphingolipids, particularly S1P, serve crucial functions in regulating the pathogenesis of *Plasmodium*, as previously reported for other microbes (31–33).

An essential part of an epigenetic indexing system for identifying transcriptionally active chromatin areas is the lysine acetylation of histones. Nuclear S1P regulates the dynamic turnover of histone acetylation states by binding to HDACs and inhibiting their functional activity, thus conferring epigenetic regulation of gene expression (15). We examined the effect of host S1P levels on *Pf*Histone-4 acetylation, and the acetylation signals were significantly reduced following PF-543 treatment. *Pf*HDAC-1 orchestrates critical biological processes involved in parasite development and infection progression (13). As a result, we investigated the impact of host S1P on *Pf*HDAC-1-mediated gene regulation. A significant decrease in the expression levels of parasite genes, previously reported to be upregulated by *Pf*HDAC inhibition (21, 36), was observed after treatment of the parasite with PF-543, which is consistent with the genome-wide transcriptional alterations in P. falciparum by HDAC inhibition (21). Particularly, the expression levels of α-tubulin were found to be severely downregulated, indicating that α-tubulin expression is a potential transcriptional marker of HDAC-1 suppression in the malarial parasite, consistent with previous findings (9, 21). *Pf*AP2-G, which is required for gametocyte maturation (39, 40), was also found to be downregulated upon PF-543 treatment. These findings indicate that inhibiting *Pf*HDAC-1 activity causes a substantial increase in global acetylation of histones and, subsequently, stimulation of basal gene transcription.

Evaluation of the gametocidal activity of hSphK-1 inhibition indicated that PF-543 attenuates early-stage gametocyte development in P. falciparum. Furthermore, we established the crucial role of host S1P during male gamete exflagellation and ookinete maturation with P. berghei ANKA-infected murine erythrocytes. *In vitro* anti-plasmodial potency of PF-543 encouraged us to evaluate it *in vivo* in mice infected with P. berghei. Interestingly, parasitemia significantly reduced to 60% in PF-543-treated mice. Moreover, the treated mice outlived the control mice by more than 12 days. These findings indicate that altering host S1P levels deregulates *Pf*HDAC-1 activity, which results in an imbalance in the global transcriptome, thus conferring transmission-blocking potential. The inhibition of P. falciparum asexual and sexual growth by the host SphK-1 inhibitor, PF-543, as shown in Fig. 3 and 8, could be attributable to abnormalities in other cellular pathways as well, including glycolysis, owing to the *pleiotropic* biological activities exerted by S1P, such as cell proliferation and survival, endothelial cell migration, maintenance of endothelial barrier integrity, and bone marrow trafficking, etc. (58, 59).

Altogether, the aforementioned findings establish host S1P as an endogenous regulator of *Pf*HDAC-1-mediated epigenetic reprogramming in the malaria parasite, and illuminate novel epigenetic mechanisms of gene regulation that might be leveraged for developing antimalarial chemotherapeutics targeting the parasite’s lipid codependency. Who would have anticipated that a humble beginning with a biochemical curiosity on an enzymatic activity extracted from the calf thymus would lead to a better understanding of epigenetics, and have the potential to impact the improvement of human health?

## MATERIALS AND METHODS

### *In vitro* culture of P. falciparum.

P. falciparum laboratory-adapted strains: 3D7 (chloroquine and artemisinin-sensitive), Dd2 (chloroquine-resistant), R539T (artemisinin-resistant), and RKL-9 (chloroquine-resistant) were cultured *in vitro* using the standard protocols, as described previously (60). Briefly, the parasites were cultured in RPMI 1640 (Gibco) medium supplemented with 5.9 gm/L HEPES (Sigma-Aldrich), 50 mg/L hypoxanthine (Sigma-Aldrich, USA), 2 gm/L sodium bicarbonate (Sigma-Aldrich), 5 gm/L AlbuMax I (for 3D7, R539T and Dd2; Gibco), or 10% heat-inactivated human serum (for RKL-9), and 10 mg/L Gentamicin (Sigma-Aldrich). All the strains were maintained in 75 cm^2^ culture flasks (Corning) using fresh O-positive (O+) human erythrocytes, under an ambient mixed gas environment (5% O_2_, 5% CO_2_, and 90% N_2_) at 37°C. Before every experiment, the parasite culture was tightly synchronized with 5% d-sorbitol for 2 successive intra-erythrocytic proliferative cycles.

### Cloning, overexpression, and purification of r*Pf*HDAC-1 and rSphK-1.

Complementarity-determining sequence (CDS) encoding full-length *Pf*HDAC-1 (1380 bp) was amplified from genomic DNA (*Pf*3D7) using the following primers: BamHI_FP: 5′-CGCGGATCCATGTCTAATAGAAAAAAGGTTGC-3′, and XhoI_RP: 5′-CCGCTCGAGTTAATATGGTACAATAGATTGATCC-3′, with Phusion High-Fidelity DNA polymerase (ThermoFisher Scientific). The amplified DNA fragment was cloned between BamHI and XhoI restriction sites of the pET-28a(+) expression vector, and the recombinant plasmid was transformed into Escherichia coli strain BL21-CodonPlus. Overexpression of 6×His-*Pf*HDAC-1 (r*Pf*HDAC-1) was induced with 0.5 mM Isopropyl-β-d-Thiogalactopyranoside (IPTG) (Sigma-Aldrich) at an optical density (OD_600_) of 0.6, for 16 h at 25°C. Bacterial cells were harvested (6000 × *g* for 15 min) and lysed by sonication in a lysis buffer containing 50 mM Na_2_HPO_4_ (pH 7.4) and 200 mM NaCl, supplemented with 500 μg/mL Lysozyme (Sigma-Aldrich), and 1 mM Phenylmethylsulfonyl Fluoride (PMSF; ThermoFisher Scientific). The bacterial lysate was centrifuged at 10,000 × *g* for 20 min, and the supernatant obtained was loaded onto a Nickel-Nitrilotriacetic Acid (Ni-NTA) agarose resin (Qiagen)-packed column along with 10 mM imidazole, for 4 h at 4°C with mild agitation. The protein was eluted with a continuous imidazole gradient of 25, 50, 100, 250, and 500 mM. The protein purification was validated by 12% SDS-PAGE, followed by immunoblotting with anti-His tag antibody.

CDS encoding full-length SphK-1 (1155 bp) was amplified using the following primers: NheI_FP: 5′-AATGCTAGCATGGATCCAGCGGGCGGCCCC-3′ and XhoI_RP: 5′-TGGCTCGAGCTATAAGGGCTCTTCTGGCGGTG-3′. The amplified DNA fragment was cloned between NheI and XhoI restriction sites of the pET-28a(+) expression vector, and the recombinant plasmid was transformed into E. coli strain BL21 (ʎDE3) gold. Overexpression of 6×His-SphK-1 (rSphK-1) was induced with 1 mM IPTG (Sigma-Aldrich) at an optical density (OD_600_) of 0.6, for 4 h at 37°C. The protein was purified using Ni-NTA agarose resin (Qiagen) by following the method described above.

### Mice immunization.

The mice were handled in accordance with the requirements of the Institutional Animal Ethics Committee (IAEC) of Jawaharlal Nehru University and the Committee for the Purpose of Control and Supervision of Experiments on Animals (CPCSEA). Three female BALB/c mice (6-weeks old) were immunized intraperitoneally (IP) with 30 μg of recombinant protein (per mouse; in 0.9% saline) mixed with an equal volume of Freund’s complete adjuvant (ThermoFisher Scientific) on day 0, followed by 2 booster regimens in Freund’s incomplete adjuvant (ThermoFisher Scientific) on days 21 and 42. Bleeds were collected from each mouse 10 days following each booster dosage. The antisera obtained from the terminal bleeds of the mice were used for all the experiments, at a dilution of 1:250.

### Microscale thermophoresis.

Binding affinities of S1P and SAHA (positive control) for r*Pf*HDAC-1 and hHDAC-1 were evaluated by MST analyses, using Monolith NT.115 instrument (NanoTemper Technologies). r*Pf*HDAC-1 (389 nM) was diluted in phosphate buffered saline (PBS; pH 7.4), supplemented with 0.05% Tween 20 (PBS-T) to prevent sample aggregation, followed by labeling with Cysteine reactive dye (30 μM; Monolith Protein Labeling Kit Red-Maleimide 2nd Generation, NanoTemper), and incubated for 30 min at room temperature (RT) in the dark. The labeled r*Pf*HDAC-1 and buffer were added to an equilibrated column, followed by collecting elution fractions. The elutes with less than 1,000 fluorescence counts were taken further for interaction analysis. Labeled r*Pf*HDAC-1was titrated with S1P and SAHA (0.01 nM to 1 mM). The samples were pre-mixed, and incubated for 10 min at RT in the dark before being loaded into standard treated capillaries (K002 Monolith NT.115). The interaction between rSphK-1 (350 nM) and PF-543 (0.01 nM to 1 mM) was also determined using MST analysis following the same protocol as mentioned above. For interaction analysis, the change in thermophoresis was expressed as the fluorescence change in MST signal, defined as F_hot_/F_cold_ (F_hot_ as the hot region after IR laser heating, and F_cold_ as the cold region at 0 s). Titration of the non-fluroescent ligand results in a gradual change in thermophoresis, which was plotted as ΔF_norm_ to yield a dose-response (binding) curve, which can be fitted to derive binding constants. Data evaluation was done with the Monolith software (Nano Temper).

### *In silico* interaction analysis.

Amino acid sequences of *Pf*HDAC-1, hHDAC-1, and hHDAC-2 (Uniprot IDs: Q9XYC7, Q13547, and Q92769, respectively) were retrieved from the UniProt database (61). For structural modeling of the proteins, Phyre2, an automatic fold recognition server for predicting the structure and/or function of a protein sequence, was used (62). Structural files of SAHA and S1P were obtained from the PubChem database (63). The structures were optimized using the Swiss PDB viewer and ChemBio Draw ultra 3D (64). The stereochemical quality of the protein structures was checked using the Procheck server (65). SAHA and S1P were docked with the generated structural models of the proteins using Autodock version 4.2 and Cygwin terminal version 3.1 (66). We ensured that the catalytic amino acid residues were covered while constructing the virtual grid for docking. For this, virtual 3D grids (spacing: 0.375; npts: 126, 126, 22; center: X-12.887, Y-22.656, Z-1.360 for *Pf*HDAC-1; spacing: 0.375; npts: 126, 126, 126; center: X-53.626, Y-12.066, Z-6.988 for hHDAC-1; and, spacing: 0.375; npts: 126, 126, 126; center: X-12.557, Y-23.038, Z-1.495 for hHDAC-2) were constructed using the Autogrid module of AutoDock Tools. Top scoring docked conformations of S1P and SAHA were selected based on their most negative free binding energies and visualized for polar contacts (if any) with the amino acid residues of *Pf*HDAC-1, hHDAC-1, and hHDAC-2 by using the PyMOL Molecular Graphics System (67). For additional analysis and visualization of the docking results, PLIP, LigPlot + version 2.2, Discovery Studio version 19.1.0, and PyMOL version 2.3.2 were used (67–69).

### Protein-lipid overlay assay.

The interaction of S1P with r*Pf*HDAC-1 was demonstrated using a protein-lipid overlay assay. S1P was solubilized in methanol:chloroform (1:1) solution and spotted on 2 nitrocellulose membranes (0.2 μm, Millipore) in different amounts (87.5, 125, 250, and 500 ng). The membranes were blocked with 3% nonfat skimmed milk in tris-buffered saline (TBS) for 1 h at RT, and washed twice with TBS-T (TBS plus 0.05% Tween 20). The membranes were incubated with r*Pf*HDAC-1 and r*Pf*Histone4 (r*Pf*H4) overnight at 4°C, followed by washing twice with TBS-T, and once with TBS. The membranes were probed with mice anti-r*Pf*HDAC-1 and mice anti-r*Pf*H4 immune sera (1:250 of each, in 1% skimmed milk in TBS-T), respectively, for 1 h at RT. After washing twice with PBS-T and once with PBS, the membranes were probed with anti-mice IgG HRP-conjugated antibody (1:5000, Sigma-Aldrich). Following a final series of 3 TBS-T washes, protein and lipid binding was detected using ECL Western blotting Substrate (Bio-Rad), and analyzed using ChemiDoc GelImaging System and Image Lab Software (Bio-Rad).

Interaction analysis between S1P and native *Pf*HDAC-1 from the parasite lysate was also done, following the same protocol described above. In particular, spots of increasing amounts (50, 100, 200, and 400 ng) of S1P were spotted on nitrocellulose membrane and air dried. A total of 200 ng of mixed phospahtidic acid (PA; 200 ng) and 500 ng of the nuclear fraction were also spotted on the membrane, as negative and positive controls, respectively. The membrane was then blocked with 5% skimmed milk followed by incubation with the parasite’s nuclear fraction (500 ng). The membrane was further probed with anti-r*Pf*HDAC-1 polyclonal antibody, and the protein and lipid binding was detected using ECL Western blotting Substrate (Bio-Rad), and analyzed using ChemiDoc Gel Imaging System and Image Lab Software (Bio-Rad).

### Pull-down assay using S1P-conjugated agarose beads.

To confirm the interaction between S1P and *Pf*HDAC-1, a Western blot-based S1P pulldown assay was performed. S1P-conjugated agarose beads were washed and equilibrated with wash buffer (10 mM HEPES [pH 7.4] and 150 mM NaCl, 0.25% NP-40) (15). Fraction 2 of the nuclear lysates, erythrocyte lysates, and r*Pf*HDAC-1 were incubated with the washed S1P agarose beads (100 μL) overnight at 4°C with constant gentle rotation. After 6 washes in wash buffer, the beads were subjected to 12% SDS–PAGE, and electrophoretically transferred to Immun-Blot polyvinylidene difluoride (PVDF) Membrane (Bio-Rad) using Trans-Blot SD Semi-Dry Transfer Cell (Bio-Rad). The blot was blocked with 5% nonfat skimmed milk in PBS-T for 1 h, followed by probing with anti-r*Pf*HDAC-1 immune sera (diluted 1:250) for 1 h. The blot was washed twice with PBS-T and once with PBS, followed by incubation with anti-mice IgG HRP-conjugated antibody (1:8000) for 1 h. After a final series of three washes, the blot was developed with ECL Western Blotting Substrate (Bio-Rad), and analyzed using ChemiDoc Gel Imaging System (Bio-Rad). Densitometry analysis was done using ImageJ software and plotted as a bar graph.

### Fluorometric *Pf*HDAC-1 enzymatic assay.

The enzymatic activity of r*Pf*HDAC-1, as well as native *Pf*HDAC-1 in the parasite’s nuclear fraction, was evaluated using the HDAC Activity assay kit (Fluorometric; Abcam, ab156064). This assay involves deacetylation of the fluoro-substrate peptide, Lys(Ac)-7-amino-4-methylcoumarin (Lys [Ac]-AMC) by HDAC, resulting in the formation of 2 hydrolyzed products: lysine and 7-amino-4-methylcoumarin (AMC). The enhanced fluorescence intensity is due to AMC (70). The following experimental groups were employed in a 96-well flat-bottom plate (Corning): no enzyme control, solvent control, S1P (2 μM)-treated, and SAHA (5 μM)-treated (positive control). The reaction was prepared by diluting 100 ng of the enzyme in 300 μL of HDAC assay buffer. In the subsequent step, 40 μL of the diluted enzyme was added to each of 10 μL of S1P (2 μM), 10 μL SAHA (5 μM), and 10 μL of solvent. The final reaction mixture (50 μL) was added to each well of the 96-well plate and incubated at 37°C for 10 min. The enzymatic reaction was initiated by adding 50 μL of Lys (Ac)-AMC, followed by incubation for 45 min at 37°C. To terminate the reaction, trypsin-containing solution (50 μL) was added to each well, and the plate was incubated at 37°C for 15 min. The enzymatic activity was assessed by measuring the fluorescence intensity of AMC using the Varioskan LUX Multimode Microplate Reader (ThermoFisher Scientific) at an excitation/emission wavelength of 360 nm/450 nm. The data obtained from 3 independent experiments were plotted as a bar graph using GraphPad Prism version 8.0.

### *In vitro* growth inhibition assay.

The *in vitro* growth inhibition of P. falciparum strains 3D7, Dd2, and R539T with PF-543 was evaluated using SYBR green I-based fluorescence assay, to determine the half-maximal effective concentration (EC_50_). A total of 100 μL of synchronized ring stage parasites of each strain at 1% parasitemia and 2% hematocrit were seeded in a 96-well flat-bottom microtiter plate (Corning). PF-543 was serially diluted and added at concentrations ranging from 0 to 8 μM, followed by incubating the parasites at 37°C for 72 h in a mixed gas environment. Artemisinin and chloroquine were used as reference compounds. Following 72 h of incubation, the parasites were lysed by freeze-thaw cycles, and 100 μL of lysis buffer (20 mM Tris-HCl, 5 mM EDTA, 0.16% [wt/vol] saponin, 1.6% [vol/vol] Triton X-100) containing 1×SYBR green I (ThermoFisher Scientific) was added to each well. Plates were incubated in the dark at RT for 3 to 4 h. Parasite proliferation was assessed by measuring the fluorescence intensity using a Varioskan LUX multimode microplate reader (ThermoFisher Scientific) with excitation and emission wavelengths of 485 nm & 530 nm, respectively. Percent growth inhibition was calculated using the formula: % Growth Inhibition = [1 – (Treated/Control)] × 100. EC_50_ values were calculated via non-linear regression analysis using GraphPad Prism version 8.0. Three independent assays each with 3 replicates were conducted in this experiment.

### Parasite invasion assay.

Uninfected erythrocytes were first treated with PF-543 (2 μM) for 2 h; untreated erythrocytes served as control. Purified *Pf*3D7 schizonts were then added to the treated, as well as untreated erythrocytes at 2% hematocrit, and incubated at 37°C. 6 h postinfection, the samples were washed with incomplete RPMI 1640 medium. Giemsa-stained smears were prepared from the samples and percent parasitemia was calculated. Following 24 h postinfection, Giemsa-stained smears were again prepared, and the number of dead parasites was counted in both untreated as well as PF-543-treated samples. The experiment was set up in duplicate wells and data from 3 independent experiments were plotted as a bar graph using GraphPad Prism version 8.0 software.

### hSphK-1 enzymatic assay using NBD-Sph.

For the hSphK-1 enzymatic assay, a fluorescent molecule: omega (7-nitro-2–1, 3-benzoxadiazol-4-yl [2S,3R,4E]-2-amino octadec-4-ene-1,3-diol [NBD–Sphingosine (NBD-Sph); Avanti Polar Lipids]) was used as a substrate, and the effect of PF-543 (2 μM) on the conversion of NBD-Sph to NBD-S1P by the parasitized erythrocytes was evaluated. Toward this, *Pf*3D7 trophozoite-infected erythrocytes (4 to 5% parasitemia) were treated with PF-543, for 4 h and 12 h. A total of 100 μL (1 × 10^8^ cells) of this parasite culture was washed with incomplete RPMI (iRPMI), and incubated with 10 μM NBD-Sph, at 37°C for 60 min. After incubation, the erythrocytes were lysed with saponin to obtain host-free parasites. The parasites were thoroughly mixed with 260 μL of methanol and 400 μL of chloroform:methanol (1:1) (71), followed by adding 16 μL of 7 M NH_4_OH, 400 μL of chloroform, and 300 μL of 1.5 M KCl. The lipids were separated by centrifugation at 17,000 × *g* for 5 min. A 100 μL aliquot of the upper (aqueous) phase was transferred to a black 96-well flat-bottom plate (Corning). Fluorescence intensity of the aqueous phase containing NBD-S1P, in PF-543 treated and untreated samples, was measured at an excitation/emission wavelength of 485 nm/530 nm using Varioskan LUX Multimode Microplate Reader (ThermoFisher Scientific). The data obtained from 3 independent experiments were plotted using GraphPad Prism version 8.0 software. To evaluate the inhibitory effect of PF-543 on the enzymatic activity of rSphK-1, the protein was pre-incubated with different concentrations of PF-543 (0 to 5 μM) for 45 min., followed by initiating the reaction in a buffer containing 0.05% Triton X-100, 1 mM ATP, 2% DMSO and NBD-Sph (10 μM). The fluorescence intensity of NBD-S1P was measured as described above.

To validate if PF-543 is not cytotoxic against P. falciparum strain 3D7 after 4 and 12 h of treatment, we determined the parasite viability using co-staining with SYTO9 Green, a membrane permeable nucleic acid stain; and, propidium iodide (PI), a red-fluorescent nuclear counterstain, which is commonly used to detect dead cells in a population. Toward this, we treated *Pf*3D7 trophozoite parasitized erythrocytes with PF-543 (2 μM), and incubated at (37°C for 4 and 12 h, respectively; untreated parasites served as control). After the specified time intervals, SYTO9/PI (1:1; 100 nM each) was added to each sample, followed by incubation in dark at 37°C for 30 min. The fluorescence intensity was measured using the Varioskan LUX Multimode Microplate Reader (ThermoFisher Scientific) at excitation/emission wavelengths of 485/498 nm (SYTO9) and 535/615 nm (PI).

### Nuclear uptake of NBD-S1P by the parasite.

Erythrocytes parasitized with *Pf*3D7 rings, trophozoites, and schizonts (1 × 10^8^/mL of each) were washed and incubated with NBD-Sph (10 μM) for 15 min at 37°C. Cells were harvested (550 × *g* for 5 min) and resuspended in iRPMI medium with 0.1% Bovine Serum Albumin ([BSA], ThermoFisher Scientific). A total of 100 μL of the cell suspension was placed in a glass bottom petri dish, and the cells were allowed to settle for 5 min. The cells were visualized using an Olympus fluorescence microscope at 100 × oil objective (Melville). From the same samples, host-free parasites were obtained by saponin lysis of erythrocytes and the uptake of NBD-S1P was detected using the same protocol as described above. To evaluate the effect of PF-543 on the uptake of NBD-S1P by the parasites, mature-stage parasites (schizonts; 1 × 10^8^/mL) were treated with PF-543 (2 μM) for 1 h, followed by incubation with NBD-Sph (10 μM) for 15 min at 37°C. The parasites were harvested, and the nuclear extract was prepared using cytoplasmic lysis buffer, the composition of which is described in the “isolation of nuclear fraction” section of the material and methods, and visualized using the Olympus fluorescence microscope. Images were captured and processed with NIS-Elements software version 4.50. The mean fluorescence intensity was plotted using GraphPad Prism version 8.0 software.

### Immunofluorescence assay to show altered histone modifications and α-tubulin expression.

To determine Histone-4 acetylation status as well as α-tubulin expression, immunofluorescence assays (IFAs) were performed on synchronized P. falciparum 3D7 cultures. *Pf*3D7 schizont stage parasites were treated individually with PF-543 (2 μM) and SAHA (5 μM; positive control) for 4 h, and untreated parasites served as control. Post-treatment, thin smears of parasites were prepared on glass slides and fixed with methanol for 30 min at –20°C. Nonspecific binding sites were blocked with 3% BSA (in PBS) for 30 min at RT. Slides were probed with mice anti-H4Kac4 antibody (1:1000; Invitrogen) and rabbit anti-α-tubulin sera (1:1000; in-house generated) at RT for 1 h. Slides were washed twice with PBS-T and once with PBS, and probed with Alexa Fluor 594 (red)-conjugated goat anti-mouse IgG and Alexa Fluor 488 (green)-conjugated goat anti-rabbit IgG antibodies (1:500; Molecular Probes) at RT for 1 h. Following washing, the slides were mounted with Hoechst stain and visualized under an Olympus fluorescence microscope. Images were processed via NIS-Elements software version 4.50. The mean fluorescence intensities were plotted using GraphPad Prism version 8.0. The experiment was performed thrice.

### Immunoblotting of altered histone modifications and α-tubulin expression.

Host-free parasites were prepared from the parasite cultures from each experimental condition used in the IFAs. Parasite pellets, thus obtained, were subjected to SDS-PAGE and electrophoretically transferred to an Immun-Blot PVDF Membrane (Bio-Rad) using a Trans-Blot SD Semi-Dry Transfer Cell (Bio-Rad). Blots were blocked with 5% nonfat skimmed milk in (PBS-T) for 1 h. To determine the histone acetylation status, blots were probed with mice anti-H4Kac4 (1:1000; Invitrogen) antibody for 1 h, and the loading control was probed with mice anti-Histone 4 (1:1000; Invitrogen) antibody. To assess the α-tubulin expression, blots were probed with rabbit anti-α-tubulin immune sera (1:1000; in-house generated), and the loading control was probed with mice anti-GAPDH antibody (1:5000; Sigma-Aldrich, USA). Following 2 PBS-T washes and 1 PBS wash, the blots were probed with anti-mice and anti-rabbit IgG HRP-conjugated antibodies (1:8000; Sigma-Aldrich) diluted in 1% skimmed milk in PBS-T for 1 h at RT. After 3 washes with PBS-T, blots were developed with ECL western Blotting Substrate (Bio-Rad), and analyzed using ChemiDoc Gel Imaging System and Image Lab Software (Bio-Rad). Densitometry analysis was done using ImageJ software and plotted as a bar graph.

### Isolation of nuclear fraction.

*Pf*3D7 nuclear fraction was extracted following the previously described methods with minor modifications (72). Briefly, erythrocytes parasitized with the mature stages of the parasite were lysed with 0.05% saponin. To collect the cytoplasmic extract (fraction 1), the parasite pellet was resuspended in a hypotonic cytoplasmic lysis buffer (CLB). Following 4 washes with CLB, the pellet was resuspended in low salt buffer (LSB) to collect fraction 2. Following 2 washes with LSB, the chromatin-containing pellet was dissolved in DNase I-digestion buffer (containing 100-500U DNase I, Sigma-Aldrich) to obtain fraction 3. After 2 washes with LSB, the pellet was solubilized in a high salt buffer (HSB) to obtain fraction 4. After 2 washes in HSB, the pellet containing the insoluble nuclear matrix was solubilized in the SDS extraction buffer to collect fraction 5. All fractions were subjected to 12% SDS-PAGE to evaluate the quality of the fractions (Fig. S6).

### Real-time quantitative reverse transcription PCR (qRT-PCR) for gene expression analysis.

P. falciparum trophozoite-infected erythrocytes were treated with PF-543 (2 μM) and SAHA (5 μM; positive control) for 3 h, and untreated parasites served as control. Total RNA was isolated using the TRIzol reagent (Invitrogen) and quantified using a Nanodrop ND-1000 spectrophotometer (ThermoFisher Scientific). Purified RNA was reverse-transcribed using an iScript cDNA synthesis kit (Bio-Rad). The resulting cDNA was diluted 1:10 with nuclease-free water, and real-time PCR analysis was performed using PowerUp SYBR Master Mix (ThermoFisher Scientific). Details of the primers used are provided in Table S1. The thermal profile used for the real-time PCR was as follows: amplification at 50°C for 2 min, followed by 40 cycles of 95°C for 15 sec, 60°C for 30 sec, and 72°C for 1 min. Melting curves were generated along with mean C_T_ values estimates, and the specific PCR product was validated. Amplification of 18S rRNA was used as an internal control for normalization. Using the 2^−ΔΔ^*^CT^*method, the results were expressed as a fold change in the expression of untreated and treated samples. Each experiment was done in triplicates and repeated three times. Statistical significance was established by Student's *t* test analysis (*, *P* < 0.05).

### *In vitro* culture of P. falciparum RKL-9 gametocytes.

Tightly synchronized *Pf*RKL-9 parasites were treated with N-Acetyl-d-Glucosamine (GlcNAc, Sigma-Aldrich) for 72 h to eliminate the asexual stage parasites. The synchronized gametocyte culture (typically at 2% to 4% gametocytaemia), thus obtained, was diluted to 1% hematocrit and seeded (100 μL per well) in 96-well flat-bottom plates (Corning,) in the presence of PF-543 at its EC_50_ (172 nM) and 2× EC_50_ values. Untreated gametocytes served as control. The culture was maintained for 12 days in complete media containing the appropriate concentrations of PF-543. Gametocytaemia was monitored daily by microscopic examination of Giemsa-stained thin blood smears, and the percent gametocytaemia was calculated using the following formula, % gametocytaemia = (Number of gametocyte /Total number of cells) ×100. The graph was plotted using GraphPad Prism version 8.0.

### P. berghei ANKA exflagellation assay.

To evaluate the effect of PF-543 on *Pb* ANKA male gametocyte exflagellation, mice (*n* = 2) were administered intraperitoneally with 30 mg/kg of phenylhydrazine (Sigma-Aldrich). Four days post-administration, mice were injected with 1.0 × 10^8^ *Pb* ANKA-infected erythrocytes via the IP route. Gametocytes were observed 3 days after the infection. After 5 days, 120 μL of the infected blood was drawn from each mouse and mixed with a complete RPMI medium. Parasitized erythrocytes were treated with PF-543 (172 nM) and incubated at 37°C for 1 h; untreated ones served as control. The blood samples were washed, and immediately mixed with 200 μL of exflagellation medium (RPMI 1640 containing 25 mM HEPES, 20% Fetal Bovine Serum, 10 mM sodium bicarbonate, and 50 mM xanthurenic acid at pH 8.0), and incubated at 20°C for 15 min (73). Exflagellation centers were counted in 25 fields at 40× magnification. The graph was plotted using GraphPad Prism version 8.0.

### P. berghei ANKA ookinete development assay.

To evaluate the effect of PF-543 on ookinete development, 1.0 × 10^8^ *Pb* ANKA-infected erythrocytes from phenylhydrazine-treated mice were collected. Parasitized RBCs were mixed with ookinete media (RPMI 1640 containing 25 mM HEPES, 20% FBS, 10 mM sodium bicarbonate, and 50 mM xanthurenic acid at pH 8.4), which included PF-543 (2 μM), while maintaining 10% hematocrit (73). The sample was incubated at 21°C for 24 h to allow for ookinete development, followed by the preparation of Giemsa-stained smears. The graph was plotted using GraphPad Prism version 8.0.

### Antimalarial activity of PF-543 *in vivo*.

*Pb* ANKA-infected donor mice were used to infect experimental BALB/c mice intraperitoneally with 0.2 mL of the infected blood (1 × 10^8^ parasitized erythrocytes). The antimalarial activity of PF-543 *in vivo* was evaluated by following the previously described protocols (74–76). Briefly, the infected experimental mice were separated into 3 groups of 3 mice each, with one group of mice receiving PF-543 (1 mg/kg) via the IP route for 3 consecutive days. Two control groups were used in parallel: one was treated with artemisinin (10 mg/kg), and the other with vehicle (DMSO), with dosages administered once a day for 3 days. Parasite proliferation and the effect of PF-543 treatment on gametocyte maturation were monitored daily by microscopic examination of Giemsa-stained thin blood smears, and parasitemia was calculated using the formula: % parasitemia = (Number of infected erythrocytes/Total number of erythrocytes) × 100. The data acquired were processed using GraphPad Prism Version 8.0.

### Schematic representation.

The schematic representation of S1P-mediated epigenetic modulation of parasite survival pathways was created with a Licensed Biorender software (BioRender.com).

### Densitometry and statistical analysis.

The immunoblot densitometry analysis was done using ImageJ (NIH) software. For normalization, band intensity of the loading control was used. Statistical analysis was performed using GraphPad Prism version 8.0. The data are expressed as the mean ± standard deviation (SD) of independent experiments (*n* = 3 to 5). The *P*-values were calculated using the Student’s *t* test, where *P* < 0.05 was considered significant. For comparing experimental groups, One-Way ANOVA along with Tukey’s multiple-comparison test was done, where *, *P < *0.05; **, *P < *0.005; ***, *P < *0.0005 considered significant. The correlation coefficient between the 2 parameters was obtained with non-linear regression analysis of log-transformed data.

### Data availability.

Raw data are available from the corresponding author upon reasonable request.
