# Supplementary material for: Host-Erythrocytic Sphingosine-1-Phosphate Regulates Plasmodium Histone Deacetylase Activity and Exhibits Epigenetic Control over Cell Death and Differentiation
Source: Microbiol Spectr. 2023 Feb 6;11(2):e02766-22. doi: 10.1128/spectrum.02766-22 (PMC10100792; doi:10.1128/spectrum.02766-22)
Supplement: Supplemental file 1 — Supplemental material. Download spectrum.02766-22-s0001.pdf, PDF file, 1.3 MB [file spectrum.02766-22-s0001.pdf]

Host-Erythrocytic Sphingosine-1-phosphate regulates *Plasmodium* histone deacetylase activity and exhibits epigenetic control over cell death and differentiation

Raj Kumar Sah[#1], Sakshi Anand[#1], Waseem Dar[#2], Ravi Jain[1], Geeta Kumari[1], Evanka Madan[1], Monika Saini[1,2], Aashima Gupta[1], Nishant Joshi[2], Rahul Singh Hada[1,2], Nutan Gupta[1], Soumya Pati[2], Shailja Singh*[1]

[1]Special Centre for Molecular Medicine, Jawaharlal Nehru University, New Delhi: 110067, India.
[2]School of Natural Sciences, Department of Life Sciences, Shiv Nadar University, Greater Noida: 201314, India.

[#] **Equal contribution**

*** Corresponding author:** Dr. Shailja Singh, Special Center for Molecular Medicine, Jawaharlal Nehru University, Delhi: 110067, India; email address: shailja.jnu@gmail.com, shailjasingh@mail.jnu.ac.in; Tel.: 011-26743038; Fax: 011-26742580; **ORCID ID: 0000-0001-5286-6605.**

Contents:
Supplementary Fig. 1-8
Supplementary Tables 1-5

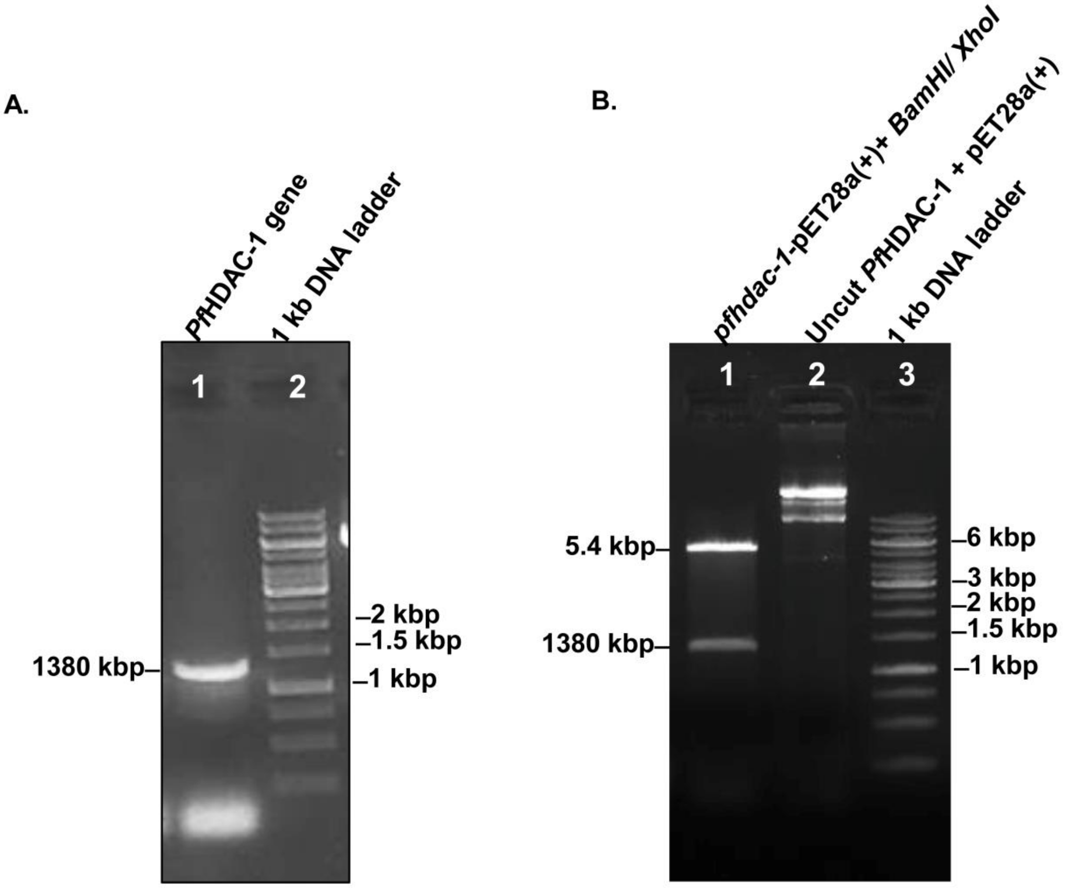

Supplementary Fig 1. Cloning of *P. falciparum hdac-1* in pET28a(+) vector. A. CDS encoding for *Pf*HDAC-1 was amplified using Phusion™ High-Fidelity DNA Polymerase. Lane 1 represents *pfhdac-1* at an expected size of 1380 bp. Lane 2 repesents 1 kb DNA ladder*. **B.** Restriction digestion of the recombinant plasmid with *BamHI/XhoI* restriction enzymes. Lane 1 represents the digested vector obtained at a size of 5.4 kbp and the fallout of *pfhdac-1* insert at 1380 bp. Lane 2 represents the uncut recombinant plasmid. Lane 3 represents 1 kb DNA ladder*. (*1 kb DNA Ladder- SM#0331, thermo Scientific GeneRuler DNA Ladder mix.)

A.

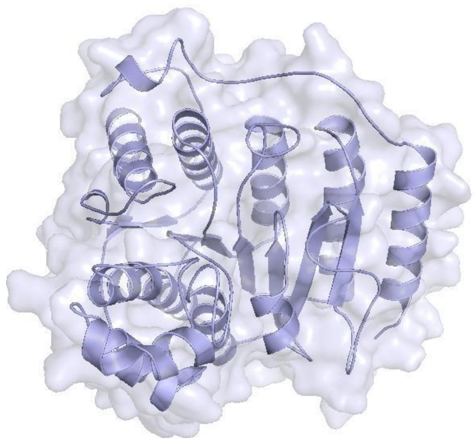

B.

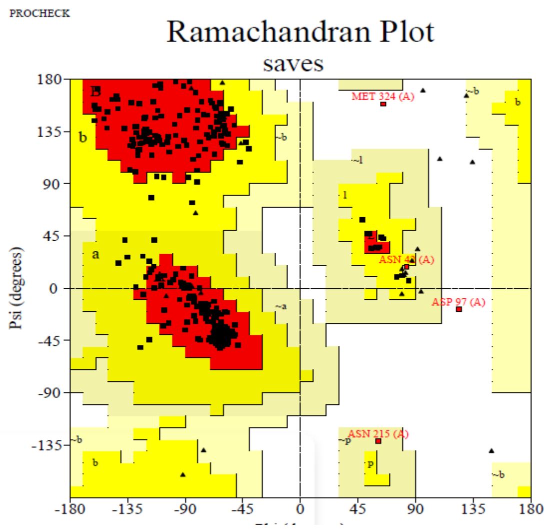

Supplementary Fig 2. Molecular Docking analysis to study protein-protein interaction. A. Energy minimized 3D protein model of *Pf*HDAC-1 used in the docking studies. **B**. Ramachandran Plot of modeled structure of *Pf*HDAC-1 depicting 90.5% amino acid residues in the core region, 8.3% amino acid residues in the allowed region, 0.6% residues in the general region and only 0.6% residues in the disallowed region.

A.

XP_001352127.1 histone deacetylase 1 [Plasmodium falciparum 3D7]

Sequence ID: **Query_24109** Length: **449** Number of Matches: **1**

Range 1: 3 to 398 Graphics   ▼ Next Match ▲ Previous Match

Score	Expect	Method	Identities	Positives	Gaps
535 bits(1379)	0.0	Compositional matrix adjust.	238/396(60%)	312/396(78%)	4/396(1%)

```
Query  7    TRRKVCYYYDGDVGNYYYGQGHPMKPHRIRMTHNLLLNYGLYRKMEIYRPHKANAEEMTK  66
             R+KV Y++D D+G+YYYG GHPMKP RIRMTH+L+++Y LY+ ME+YRPHK++  E+T
Sbjct  3    NRKKVAYFHDPDIGSYYYGAGHPMKPQRIRMTHSLIVSYNLYKYMEVYRPHKSDVNELTL  62

Query  67   YHSDDYIKFLRSIRPDNMSEYSKQMQRFNVGE--DCPVFDGLFEFCQLSTGGSVASAVKL  124
            +H  +YI FL SI  +N  E++ Q++RFNVGE  DCPVFDGLF+F Q   G S+  A KL
Sbjct  63   FHDYEYIDFLSSISLENYREFTYQLKRFNVGEATDCPVFDGLFQFQQSCAGASIDGASKL  122

Query  125  NKQQTDIAVNWAGGLHHAKKSEASGFCYVNDIVLAILELLKYHQRVLYIDIDIHHGDGVE  184
            N    DI VNW+GGLHHAK SEASGFCY+NDIVL ILELLKYH RV+YIDID+HHGDGVE
Sbjct  123  NHHCADICVNWSGGLHHAKMSEASGFCYINDIVLGILELLKYHARVMYIDIDVHHGDGVE  182

Query  185  EAFYTTDRVMTVSFHKYGEYFPGTGDLRDIGAGKGKYYAVNYPLRDGIDDESYEAIFKPV  244
            EAFY T RVMTVSFHK+G+YFPGTGD+ D+G   GKYY+VN PL DG+ D+++  +FK V
Sbjct  183  EAFYVTHRVMTVSFHKFGDYFPGTGDITDVGVNHGKYYSVNVPLNDGMTDDAFVDLFKVV  242

Query  245  MSKVMEMFQPSAVVLQCGSDSLSGDRLGCFNLTIKGHAKCVEFVKSFNLPMLMLGGGGYT  304
            + K ++ ++P A+++QCG+DSL+GDRLG FNLTIKGHA+CVE V+S+N+P+L+LGGGGYT
Sbjct  243  IDKCVQTYRPGAIIIQCGADSLTGDRLGRFNLTIKGHARCVEHVRSYNIPLLVLGGGGYT  302

Query  305  IRNVARCWTYETAVALDT--EIPNELPYNDYFEYFGPDFKLHISPSNMTNQNTNEYLEKI  362
            IRNV+RCW YET V L+   E+P+++  NDY++Y+ PDF+LH+ PSN+ N N+ E+L +I
Sbjct  303  IRNVSRCWAYETGVVLNKHHEMPDQISLNDYYDYYAPDFQLHLQPSNIPNYNSPEHLSRI  362

Query  363  KQRLFENLRMLPHAPGVQMQAIPEDAIPEESGDEDE  398
            K ++ ENLR + HAPGVQ   +P D    +  DE +
Sbjct  363  KMKIAENLRHIEHAPGVQFSYVPPDFFNSDIDDESD  398
```

B.

XP_001347363.1 histone deacetylase 2 [Plasmodium falciparum 3D7]

Sequence ID: **Query_37627** Length: **2379** Number of Matches: **2**

Range 1: 1167 to 1279 Graphics   ▼ Next Match ▲ Previous Match

Score	Expect	Method	Identities	Positives	Gaps
55.8 bits(133)	1e-11	Compositional matrix adjust.	30/113(27%)	57/113(50%)	5/113(4%)

```
Query  204   YFPGTGDLRDIGAGKGKYYAVNYPLRDGIDDESYEAIFKPVMSKVMEMFQPSAVVLQCGS  263
             ++P TG   ++G  +G  + +N PL  G ++      +FK ++  ++E F+P  + + CG
Sbjct  1167  FYPRTGAKNELGEKEGYKFNINVPLEKGYNNCDVYYVFKYLLLPILEKFRPEFIFISCGF  1226

Query  264   DSLSGDRLGCFNLTIKGHAKCVEFVKSF-----NLPMLMLGGGGYTIRNVARC  311
             D+   D LG  NLT    +       +K F     N  ++++   GGY +   + +C
Sbjct  1227  DASINDPLGKCNLTHNLYQWMTFQLKHFANIFCNGRIILVLEGGYNLNYLPKC  1279
```

Range 2: 956 to 1033 Graphics   ▼ Next Match ▲ Previous Match ▲ First Match

Score	Expect	Method	Identities	Positives	Gaps
52.4 bits(124)	1e-10	Compositional matrix adjust.	27/78(35%)	41/78(52%)	5/78(6%)

```
Query  129   TDIAVNWAGGL---HHAKKSEASGFCYVNDIVLAILELLKYH--QRVLYIDIDIHHGDGV  183
             TDI   +A       HH  +S  SGFC  N+I +A   + K +  ++V   D D+HH +G
Sbjct  956   TDINCGFAAIRPPGHHCSRSHPSGFCIFNNISVACKYIFKKYGIRKVFIFDWDVHHDNGT  1015

Query  184   EEAFYTTDRVMTVSFHKY  201
             +E FY    V+  S H++
Sbjct  1016  QEIFYGDKDVLCFSIHRF  1033
```

C.

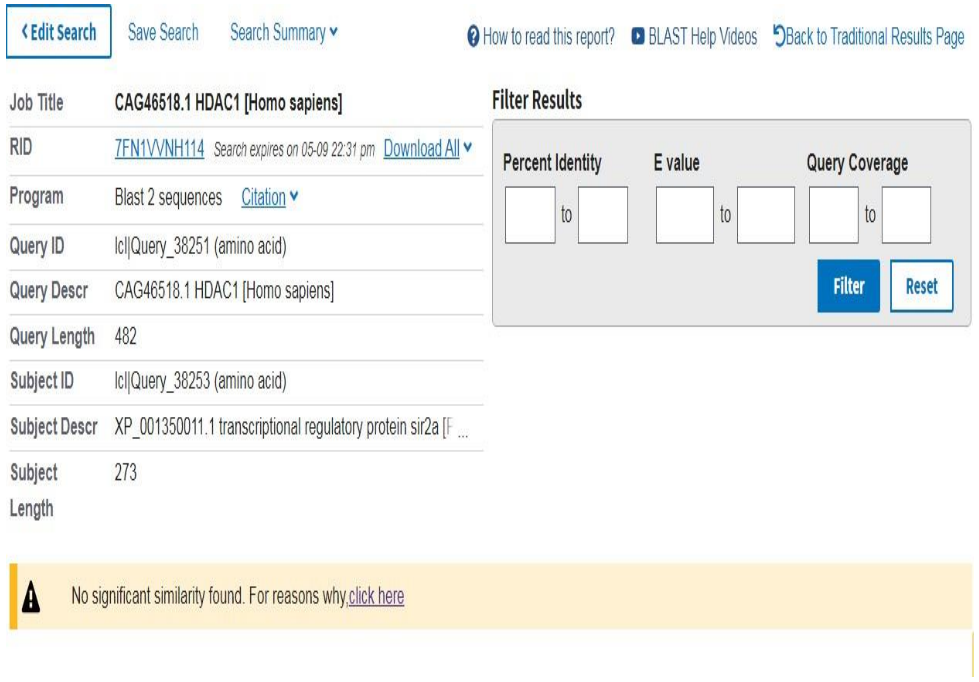

D.

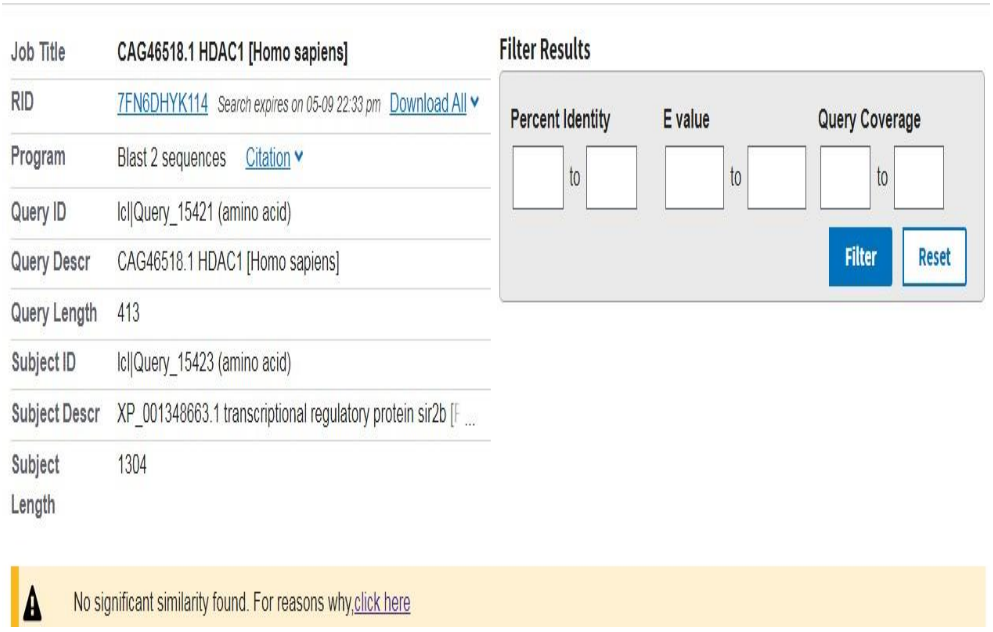

Supplementary Fig 3. Sequence alignment data of hHDAC-1 with *Pf* HDAC isoforms. A. sequence alignment between hHDAC-1 and *Pf*HDAC-1 shows 60% sequence identity. **B.** Sequence alignment between hHDAC-1 and *Pf*HDAC-2 shows 27% sequence identity. **C-D.** Sequence alignment of hHDAC-1 with *Pf*Sir2a and *Pf*Sir2b shows no significant similarity.

A.

XP_001352127.1 histone deacetylase 1 [Plasmodium falciparum 3D7]

Sequence ID: **Query_62009** Length: **449** Number of Matches: **1**

Range 1: 4 to 396 Graphics ▼ Next Match ▲ Previous Match

Score	Expect	Method	Identities	Positives	Gaps
542 bits(1396)	0.0	Compositional matrix adjust.	241/393(61%)	313/393(79%)	4/393(1%)

```
Query  9    KKKVCYYYDGDIGNYYYGQGHPMKPHRIRMTHNLLLNYGLYRKMEIYRPHKATAEEMTKY  68
            +KKV Y++D DIG+YYYG GHPMKP RIRMTH+L+++Y LY+ ME+YRPHK+   E+T +
Sbjct  4    RKKVAYFHDPDIGSYYYGAGHPMKPQRIRMTHSLIVSYNLYKYMEVYRPHKSDVNELTLF  63

Query  69   HSDEYIKFLRSIRPDNMSEYSKQMQRFNVGE--DCPVFDGLFEFCQLSTGGSVAGAVKLN  126
            H  EYI FL SI  +N  E++ Q++RFNVGE  DCPVFDGLF+F Q   G S+ GA KLN
Sbjct  64   HDYEYIDFLSSISLENYREFTYQLKRFNVGEATDCPVFDGLFQFQQSCAGASIDGASKLN  123

Query  127  RQQTDMAVNWAGGLHHAKKSEASGFCYVNDIVLAILELLKYHQRVLYIDIDIHHGDGVEE  186
                D+ VNW+GGLHHAK SEASGFCY+NDIVL ILELLKYH RV+YIDID+HHGDGVEE
Sbjct  124  HHCADICVNWSGGLHHAKMSEASGFCYINDIVLGILELLKYHARVMYIDIDVHHGDGVEE  183

Query  187  AFYTTDRVMTVSFHKYGEYFPGTGDLRDIGAGKGKYYAVNFPMRDGIDDESYGQIFKPII  246
            AFY T RVMTVSFHK+G+YFPGTGD+ D+G   GKYY+VN P+ DG+ D+++  +FK +I
Sbjct  184  AFYVTHRVMTVSFHKFGDYFPGTGDITDVGVNHGKYYSVNVPLNDGMTDDAFVDLFKVVI  243

Query  247  SKVMEMYQPSAVVLQCGADSLSGDRLGCFNLTVKGHAKCVEVVKTFNLPLLMLGGGGYTI  306
             K ++ Y+P A+++QCGADSL+GDRLG FNLT+KGHA+CVE V+++N+PLL+LGGGGYTI
Sbjct  244  DKCVQTYRPGAIIIQCGADSLTGDRLGRFNLTIKGHARCVEHVRSYNIPLLVLGGGGYTI  303

Query  307  RNVARCWTYETAVALD--CEIPNELPYNDYFEYFGPDFKLHISPSNMTNQNTPEYMEKIK  364
            RNV+RCW YET V L+   E+P+++  NDY++Y+ PDF+LH+ PSN+ N N+PE++ +IK
Sbjct  304  RNVSRCWAYETGVVLNKHHEMPDQISLNDYYDYYAPDFQLHLQPSNIPNYNSPEHLSRIK  363

Query  365  QRLFENLRMLPHAPGVQMQAIPEDAVHEDSGDE  397
             ++ ENLR + HAPGVQ   +P D  + D  DE
Sbjct  364  MKIAENLRHIEHAPGVQFSYVPPDFFNSDIDDE  396
```

B.

XP_001347363.1 histone deacetylase 2 [Plasmodium falciparum 3D7]

Sequence ID: **Query_56541** Length: **2379** Number of Matches: **2**

Range 1: 1167 to 1279 Graphics ▼ Next Match ▲ Previous Match

Score	Expect	Method	Identities	Positives	Gaps
55.1 bits(131)	2e-11	Compositional matrix adjust.	28/113(25%)	57/113(50%)	5/113(4%)

```
Query  205   YFPGTGDLRDIGAGKGKYYAVNFPMRDGIDDESYGQIFKPIISKVMEMYQPSAVVLQCGA  264
             ++P TG   ++G  +G  + +N P+  G ++      +FK ++  ++E ++P  + + CG
Sbjct  1167  FYPRTGAKNELGEKEGYKFNINVPLEKGYNNCDVYYVFKYLLLPILEKFRPEFIFISCGF  1226

Query  265   DSLSGDRLGCFNLTVKGHAKCVEVVKTF-----NLPLLMLGGGGYTIRNVARC  312
             D+   D LG  NLT   +       +K F     N  ++++  GGY +  + +C
Sbjct  1227  DASINDPLGKCNLTHNLYQWMTFQLKHFANIFCNGRIILVLEGGYNLNYLPKC  1279
```

Range 2: 970 to 1033 Graphics ▼ Next Match ▲ Previous Match ▲ First Match

Score	Expect	Method	Identities	Positives	Gaps
51.2 bits(121)	2e-10	Compositional matrix adjust.	23/64(36%)	36/64(56%)	2/64(3%)

```
Query  141   HHAKKSEASGFCYVNDIVLAILELLKYH--QRVLYIDIDIHHGDGVEEAFYTTDRVMTVS  198
             HH  +S  SGFC  N+I +A   + K +  ++V   D D+HH +G +E FY    V+  S
Sbjct  970   HHCSRSHPSGFCIFNNISVACKYIFKKYGIRKVFIFDWDVHHDNGTQEIFYGDKDVLCFS  1029

Query  199   FHKY  202
              H++
Sbjct  1030  IHRF  1033
```

C.

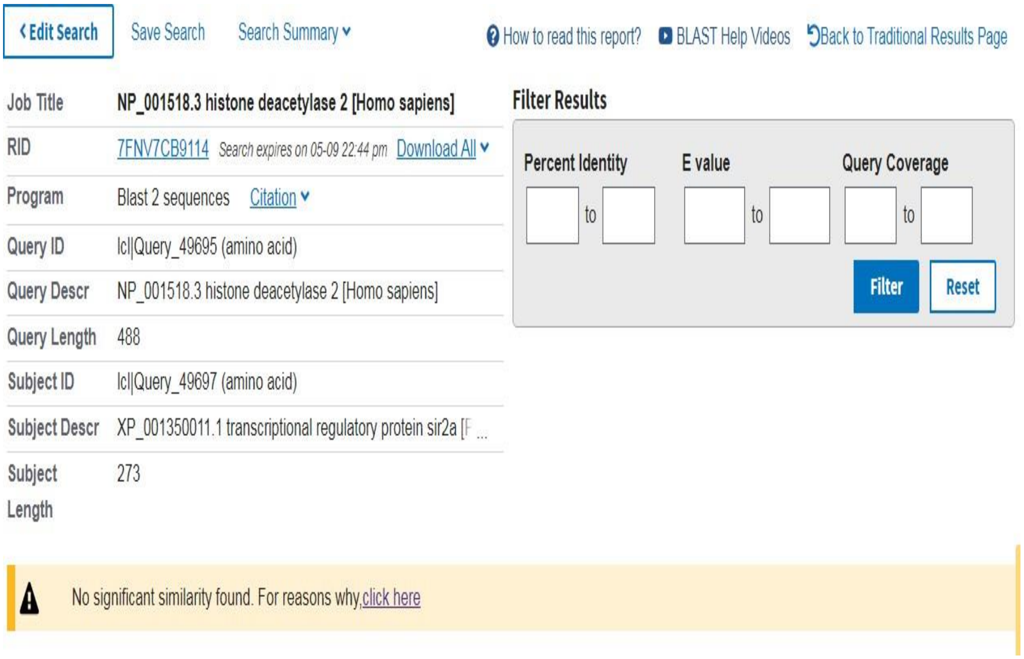

D.

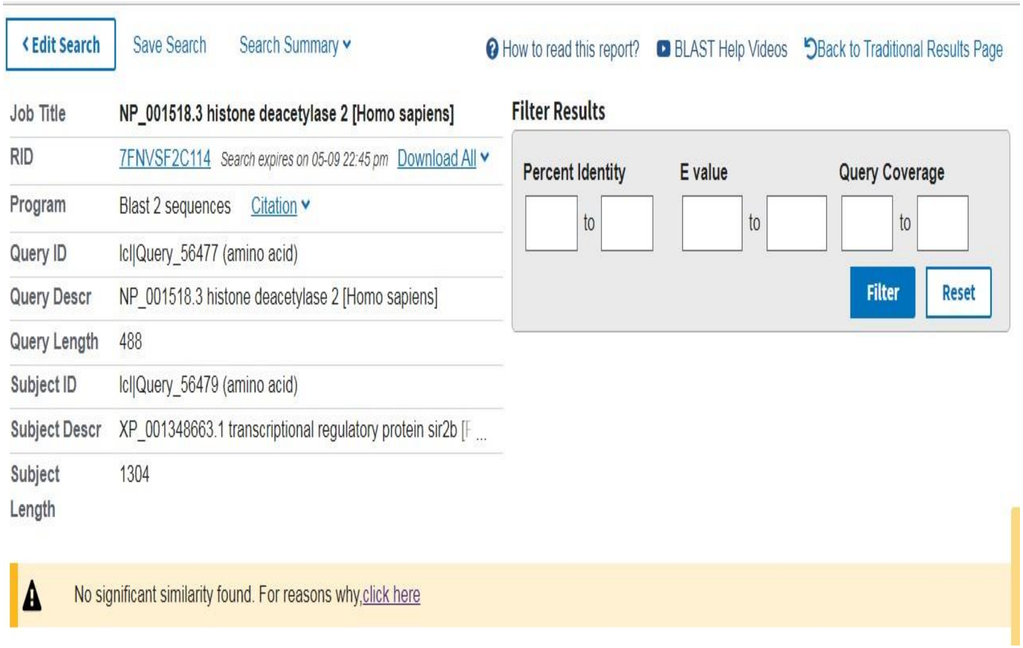

Supplementary Fig 4. Sequence alignment data of human HDAC-2 with *Pf* HDAC isoforms. A. Sequence alignment between hHDAC-2 and *Pf*HDAC-1 shows 61% sequence identity. **B.** Sequence alignment between hHDAC-2 and *Pf*HDAC-2 shows 25% sequence identity. **C-D.** Sequence alignment of hHDAC-2 with *Pf*Sir2a and *Pf*Sir2b shows no significant similarity.

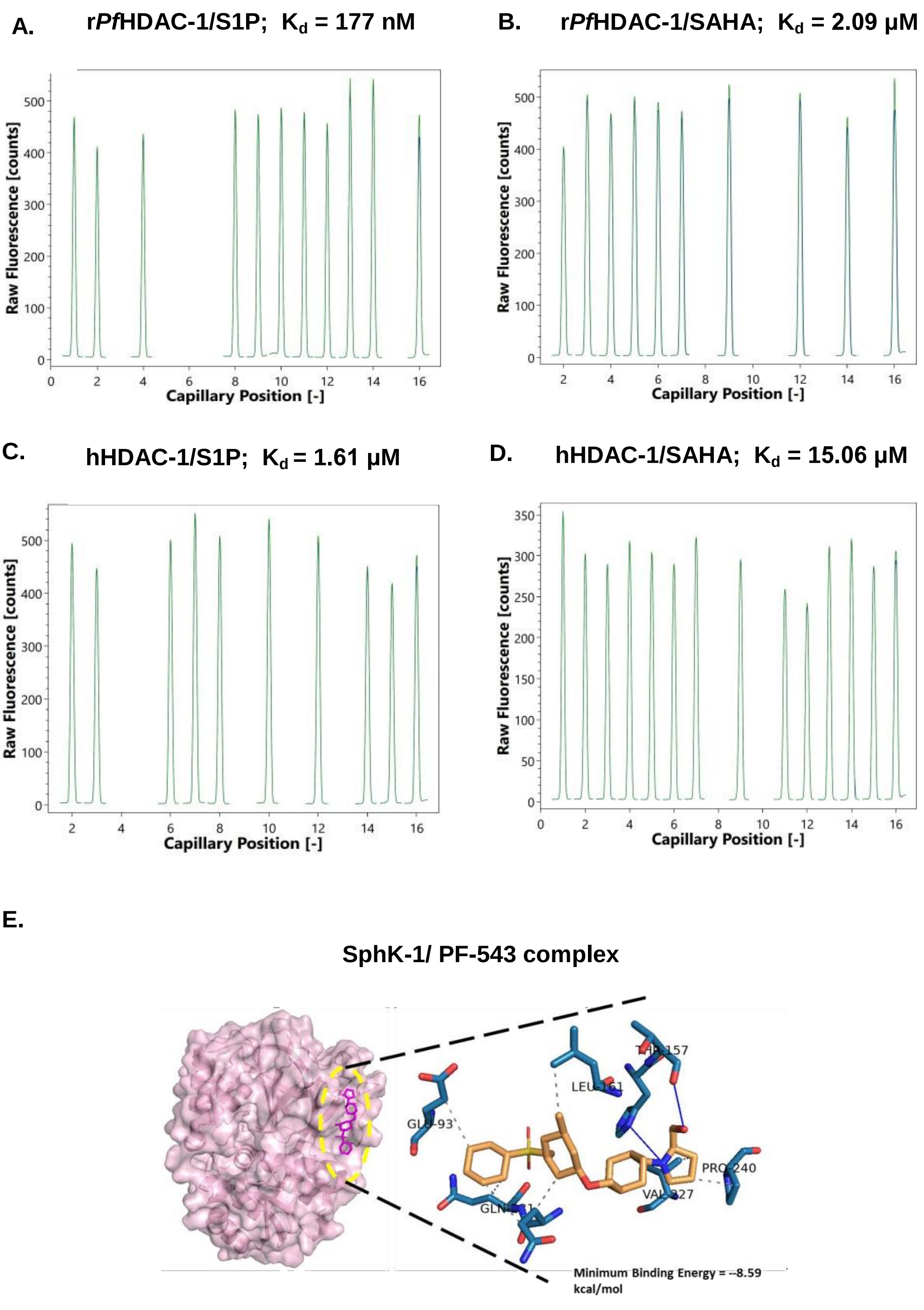

Supplementary Fig 5. Capillary scan profile of MST analysis of labelled **A.** *Pf*HDAC-1 with S1P **B.** *Pf*HDAC-1 with SAHA **C.** hHDAC-1 with S1P and **D.** hHDAC-1 with SAHA. **E.** Molecular docking analysis of SphK-1 with its potent inhibitor PF-543.

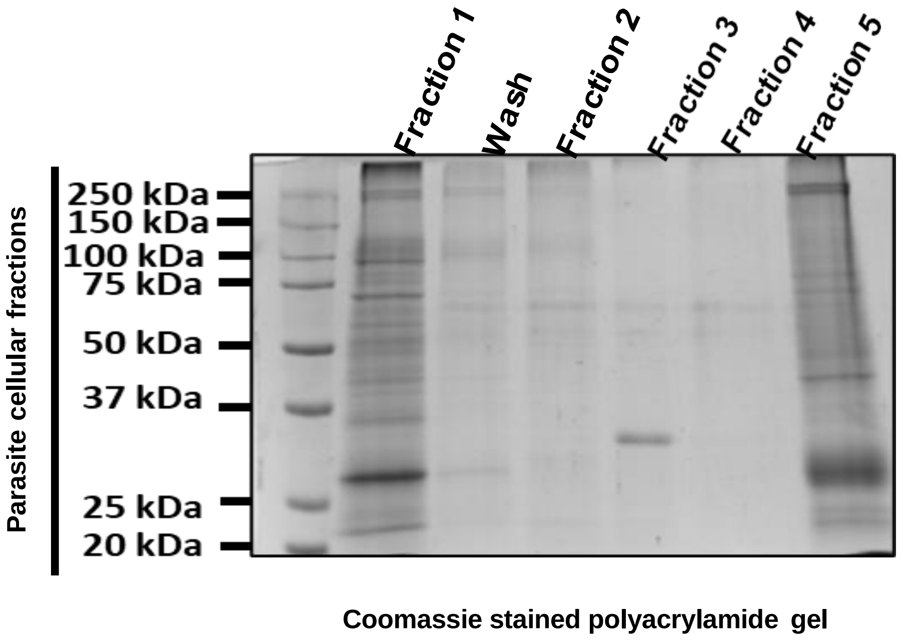

Supplementary Fig 6. Coomassie stained polyacrylamide gel showing proteins in the *Pf*3D7 parasite cellular fractions.

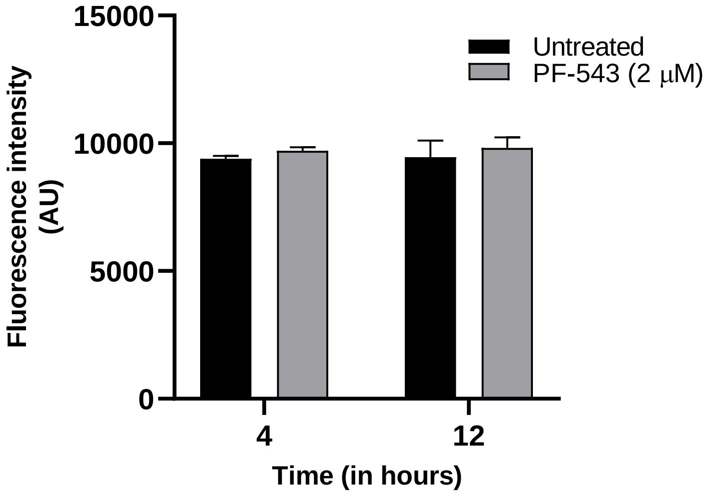

Supplementary Fig 7. Cell viability assay was performed using SYTO-9/PI (1:1) staining. *Pf*3D7 parasites were treated with PF-543 (2 µM) for 4 h and 12 h intervals and the untreated parasites were taken as control. Post-treatment, the parasites were subjected to SYTO-9/PI staining to determine their viability. As shown in the graph, the viability of the cells was not compromised even at 12 h post-treatment with PF-543 (2 µM). Additionally, the cells were found to be PI negative and SYTO-9 positive.

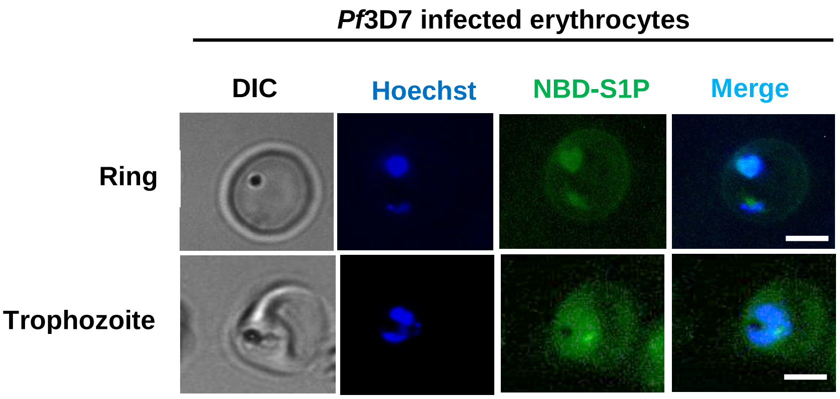

Supplementary Figure 8: A. Fluorescence microscopic images showing the uptake of NBD-Sph by *Pf*3D7 ring and trophozoite-infected erythrocytes. The presence of NBD-S1P (green) in the parasite nuclei is depicted by its co-localization with Hoechst nuclear stain (blue). Scale bar: 5 μm

S.No.	Target genes	Forward Primer	Reverse Primer	Annealing Temp. (°C)
1.	α-tubulin 2 (PF3D7_0422300)	5′-TGAACATGGAATTCAACCGG-3′	5′- CGTCAACGACGGTGGGTTC-3′	61
2.	CPPUF (PF3D7_1146800)	5' TTCTTCCTCTCTTGAGCATTCC3'	5' TTTCATGTTGCTTTGATTTACG3'	61
3.	ETMP4 (PF3D7_0423700)	5' TCTTAGGTAGTGCTTTAGGTTTTGG3'	5' TGCTTTCATCTTTTTCGTCACC3'	61
4.	PFMC-2TMI (PF3D7_0101300)	5' TTTGGATTGTTTCAACGACCT3'	5' CATGTCAGGAAAATAACGAGCA3'	61
5.	PFMC-2TMII (PF3D7_0222100)	5' ATTTGGATTGTTTCAACGACCT3'	5' CATGTCAGGAAAATAACGAGCA3'	61
6.	GA27/25 (PF3D7_1302100)	5' GCCCTTGGATAAATTTGGAA3'	5' GGATCCTTGCTAAGGGTCATC3'	61
7.	PVMPS16 (PF3D7_0406200)	5' TTCTTCGCTTTTGCAAACCT3'	5' AAAGGCATTTTGTCAGCAGAA3'	61
8.	MDG1 (PF3D7_1216500)	5' TAGGAGCAAAAGCAGGTGAT3'	5' CCGTTTCTTCATTAGCATTTCC3'	61
9.	PHISTB (PF3D7_1372100)	5' TGAAGATACGCACTTGGATGA3'	5' TCTTTCAATTTTCCCACATCG3'	61
10.	CPPUF(1) (PF3D7_1467600)	5' GAAGCAAAAACGTGGACCAT3'	5' TGACGTCTTTCCCTTTTGCT3'	61
11.	6-CP47 (PF3D7_1346800)	5' TTTAATCCCCTGACTAATGTTAAGC3'	5' ACTTCTTCGTATATTTCAGATGACC 3'	61
12.	AT2 (PF3D7_0422300)	5' ATGCAGTAGGTGGAGGTACAGG3'	5' CTGTCGATACTTGAGGAGATGG3'	61
13.	ETMP10.3 (PF3D7_1016900)	5' ATGAAGGTTTCTAGGCATACCG3'	5' AACGCTCTCTTATCATCATTTGC3'	61
14.	PFEMP1-I (PF3D7_0324900)	5' TAGCCTGTGATGATTTTGAACC3'	5' GACGTTTCTTTTGTTGTTGTTCC3'	61
15.	PFEMP1-II (PF3D7_0833500)	5' GTCCCCACATATTTCGACTACG3'	5' ACAAAAATCTTCTGCCCATTCC3'	61
16.	H4 (PF3D7_1105000)	5' GGTAAGGGAGGTAAAGGTTTGG3'	5' CTTCTTGCTAAACGTCTGATGG3'	61
17.	18S (PF3D7_1148600)	5'- CCGCCCGTCGCTCCTACCG-3'	5'-CCTTGTTACGACTTCTCCTTCC-3'	55-60
18.	AP-2 (PF3D7_1222600)	5'-GTTGGTGGTGTTTGCTTTGA-3'	5'-TCCATCGATTTCTTGGCTGT-3'	61

Supplementary Table 1. Table showing the primer sequences of the gene sets which were studied for their regulation by *Pf*HDAC-1 and SAHA through quantitative RT-PCR. The primer sequence of 18s rRNA gene (positive control) has also been mentioned in the table.

**Hydrophobic Interactions**							
**Index**	**Residue**	**AA**	**Distance**	**Ligand Atom**	**Protein Atom**		
1	19A	TYR	3.66	3003	137		
2	19A	TYR	3.36	3006	135		
3	22A	ALA	3.27	3002	160		
4	77A	LEU	3.6	3008	629		
5	94A	GLU	3.42	3013	782		
6	99A	PRO	3.69	3013	816		
7	101A	PHE	3.14	3010	830		
**Hydrogen Bonds**							
**Index**	**Residue**	**AA**	**Distance H-A**	**Distance D-A**	**Donor Angle**	**Donor Atom**	**Acceptor Atom**
1	22A	ALA	2.09	2.93	143.57	3024 [O3]	159 [O2]
2	22A	ALA	1.92	2.81	152.07	3022 [O3]	159 [O2]
3	94A	GLU	1.68	2.61	168.62	784 [O3]	3015 [N3]
4	94A	GLU	1.83	2.61	130.16	3015 [N3]	784 [O3]

Supplementary Table 2. Table representing the amino acid residues involved in establishing the interaction between S1P and *Pf*HDAC-1**.**

Hydrophobic Interactions							
Index	Residue	AA	Distance	Ligand Atom	Protein Atom		
1	185B	GLU	3.15	3684	1797		
2	188B	TYR	3.35	3684	1824		
3	188B	TYR	3.33	3687	1827		
**Hydrogen Bonds**							
**Index**	**Residue**	**AA**	**Distance H-A**	**Distance D-A**	**Donor Angle**	**Donor Atom**	**Acceptor Atom**
1	144B	LYS	3.09	4.03	153.58	1394 [Nam]	3659 [O3]
2	145B	SER	3.06	3.87	136.84	1407 [Nam]	3671 [O3]
3	185B	GLU	1.9	2.82	166.49	1800 [O3]	3659 [O3]
4	185B	GLU	2.54	3.08	116.71	3671 [O3]	1800 [O3]
5	185B	GLU	1.62	2.59	153.94	3662 [N3]	1800 [O3]

Supplementary Table 3. Table representing the critical amino acid residues involved in establishing the interaction between S1P and hHDAC-1.

Hydrophobic Interactions							
Index	Residue	AA	Distance	Ligand Atom	Protein Atom		
1	155A	PHE	3.88	3702	1509		
2	209A	TYR	3.73	3706	2048		
3	209A	TYR	3.35	3705	2050		
4	210A	PHE	3.35	3706	2059		
5	210A	PHE	3.74	3700	2060		
6	210A	PHE	3.88	3701	2061		
7	276A	LEU	3.03	3702	2670		
**Hydrogen Bonds**							
**Index**	**Residue**	**AA**	**Distance H-A**	**Distance D-A**	**Donor Angle**	**Donor Atom**	**Acceptor Atom**
1	100A	ASN	2.22	3.2	159.99	1014 [Nam]	3691 [O2]
2	104A	ASP	2.59	3.42	137.02	3685 [N3]	1047 [O2]
3	104A	ASP	2.26	3.09	144.2	3682 [O3]	1048 [O3]
4	104A	ASP	2.3	2.8	112.82	1048 [O3]	3694 [O3]
5	104A	ASP	1.93	2.8	151.92	3694 [O3]	1048 [O3]
6	153A	SER	2.12	2.91	140.82	3692 [O3]	1491 [O3]
7	154A	GLY	1.94	2.84	145.97	1493 [Nam]	3694 [O3]

Supplementary Table 4. Table representing the critical amino acid residues involved in establishing the interaction between S1P and hHDAC-2.

Hydrophobic Interactions							
Index	Residue	AA	Distance	Ligand Atom	Protein Atom		
1	148A	PHE	3.14	3671	1446		
2	148A	PHE	3.65	3672	1445		
3	202A	TYR	3.09	3666	1980		
4	203A	PHE	3.92	3659	1992		
5	203A	PHE	3.04	3665	1989		
6	203A	PHE	3.36	3670	1993		
7	269A	LEU	3.58	3669	2592		
8	269A	LEU	3.13	3668	2590		
9	301A	TYR	3.77	3673	2896		
**Hydrogen Bonds**							
Index	Residue	AA	Distance H-A	Distance D-A	Donor Angle	Donor Atom	Acceptor Atom
1	138A	HIS	2.32	3.33	171.92	1355 [Npl]	3676 [O2]
2	139A	HIS	2.83	3.65	137.2	1368 [Npl]	3676 [O2]
3	147A	GLY	2.33	3.3	159.22	3677 [Nam]	1434 [O2]
4	174A	ASP	2.64	3.55	163.09	3679 [O3]	1702 [O2]
5	176A	HIS	2.04	2.91	141.56	1719 [Npl]	3676 [O2]
6	299A	GLY	3.62	4.02	105.22	2877 [Nam]	3676 [O2]
7	301A	TYR	2.72	3.67	176.89	2899 [O3]	3676 [O2]
**π-Stacking**							
Index	Residue	AA	Distance	Angle	Offset	Stacking Type	Ligand Atoms
1	176A	HIS	4.55	63.27	0.85	T	3664, 3665, 3666, 3667, 3668, 3669

Supplemetary Table 5 :- Table representing the critical amino acid residues involved in establishing the interaction between SAHA and *Pf*HDAC-1.

e
